# Thermodynamic modeling of genome-wide nucleosome depleted regions in yeast

**DOI:** 10.1371/journal.pcbi.1008560

**Published:** 2021-01-11

**Authors:** Hungyo Kharerin, Lu Bai

**Affiliations:** 1 Department of Biochemistry and Molecular Biology, The Pennsylvania State University, University Park, Pennsylvania, United States of America; 2 Center for Eukaryotic Gene Regulation, The Pennsylvania State University, University Park, Pennsylvania, United States of America; 3 Department of Physics, The Pennsylvania State University, University Park, Pennsylvania, United States of America; Rutgers University, UNITED STATES

## Abstract

Nucleosome positioning in the genome is essential for the regulation of many nuclear processes. We currently have limited capability to predict nucleosome positioning *in vivo*, especially the locations and sizes of nucleosome depleted regions (NDRs). Here, we present a thermodynamic model that incorporates the intrinsic affinity of histones, competitive binding of sequence-specific factors, and nucleosome remodeling to predict nucleosome positioning in budding yeast. The model shows that the intrinsic affinity of histones, at near-saturating histone concentration, is not sufficient in generating NDRs in the genome. However, the binding of a few factors, especially RSC towards GC-rich and poly(A/T) sequences, allows us to predict ~ 66% of genome-wide NDRs. The model also shows that nucleosome remodeling activity is required to predict the correct NDR sizes. The validity of the model was further supported by the agreement between the predicted and the measured nucleosome positioning upon factor deletion or on exogenous sequences introduced into yeast. Overall, our model quantitatively evaluated the impact of different genetic components on NDR formation and illustrated the vital roles of sequence-specific factors and nucleosome remodeling in this process.

## Author summary

Nucleosome is the basic unit of chromatin, containing 147 base-pairs of DNA wrapped around a histone core. The positioning of nucleosomes, i.e., which parts of DNA are inside nucleosome and which parts are nucleosome-free, is highly regulated. In particular, regulatory sequences tend to be exposed in nucleosome-depleted regions (NDRs), and such exposure is crucial for a variety of processes including DNA replication, repair, and gene expression. Here, we used a thermodynamics model to predict nucleosome positioning on the yeast genome. The model shows that the intrinsic sequence preference of histones is not sufficient in generating NDRs. In contrast, binding of a few transcription factors, especially RSC, is largely responsible for NDR formation. Nucleosome remodeling activity is also required in the model to recapitulate the NDR sizes. This model contributes to our understanding of the mechanisms that regulate nucleosome positioning. It can also be used to predict nucleosome positioning in mutant yeast or on novel DNA sequences.

## Introduction

DNA wraps around histone octamers to form nucleosomes, an essential unit for chromatin organization in the nucleus [1,2]. The positioning of nucleosomes in yeast is generally characterized by well-positioned nucleosome arrays in the genic regions and low nucleosome density in the intergenic regions, with the latter often referred to as “nucleosome depleted regions (NDRs)” [3–6]. These open regions of chromatin allow regulatory factors to access DNA, which is critical for genomic processes such as transcription, replication, and DNA repair [7–10]. The NDRs may also affect the mechanical properties of chromosomes and their 3D organization [11]. Elucidating nucleosome and NDR configurations thus represent a key step towards understanding chromatin and genome functions.

While many techniques, including MNase-seq, DNaseI-seq, and ATAC-seq, are routinely used to map the NDRs over the genome, we need a more fundamental and mechanistic understanding of how these structures are formed. Recent studies have shown that multiple factors contribute to NDR formation. The intrinsic bendability of DNA sequence can affect the stability of nucleosomes [12,13], and rigid DNA sequences, like poly(A/T) stretch, disfavor nucleosome formation [14]. Consistently, *in vitro* chromatin reconstitution experiments with purified histones and DNA can partially recapitulate the nucleosome positioning *in vivo* [3,15]. However, these experiments also revealed that the intrinsic nucleosome stability is insufficient in determining the nucleosome positioning *in vivo*. Inside cells, a subset of sequence-specific transcription factors (TFs) called pioneer factors (PFs) can invade and displace nucleosomes to generate NDRs [16–18]. Nucleosome positioning also critically depends on nucleosome remodeling complexes [19]. Using energy derived from ATP hydrolysis, these complexes can slide nucleosomes along DNA and/or evict histones [20,21]. Disruption of these enzymes causes global nucleosome repositioning. In particular, deletion of RSC, an essential SWI/SNF-family remodeler in budding yeast, causes the shrinkage of a large fraction of NDRs [22,23].

Even though many genetic components are known to contribute to NDR formation, their relative importance in this process is not clear. One way to evaluate this issue is to build a mathematical model and examine the contribution from each component quantitatively. A number of nucleosome positioning models have been put forward in recent years [3,15,24,25]. Most of these models focus on the intrinsic histone preference and have limited success in predicting NDRs found *in vivo*. A recent study considering TF binding showed significantly enhanced predictability of NDRs [26]. However, the prediction of NDR location and size requires further improvement.

In this work, we present a statistical mechanics model to predict genome-wide nucleosome positioning in budding yeast by incorporating the intrinsic affinity of histones, competitive binding of sequence-specific factors, and nucleosome remodeling. We found that the binding of a few TFs, especially the binding of RSC on GC-rich and poly(A/T) sequences, allow us to predict 66% of genome-wide NDRs with a false positive rate of 0.05. In contrast, the consideration of the intrinsic histone affinity and the binding cooperativity between TFs do not significantly improve the NDR predictability. The NDRs generated by the physical exclusion of TFs are predicted to be shorter than those measured *in vivo*, which was rectified by incorporating TF-recruited nucleosome remodeling activities. We further tested the model by predicting nucleosome positioning in the absence of certain TFs or on exogenous sequences. Close agreement between the prediction and the corresponding experimental data provides strong support for our model. Finally, our model outperforms several other related models, showing that incorporation of TFs and remodeling activity improves our understanding of the *in vivo* nucleosome positioning.

## Results

### A thermodynamic model of TF-chromatin association to predict nucleosome positioning

**Fig 1:**
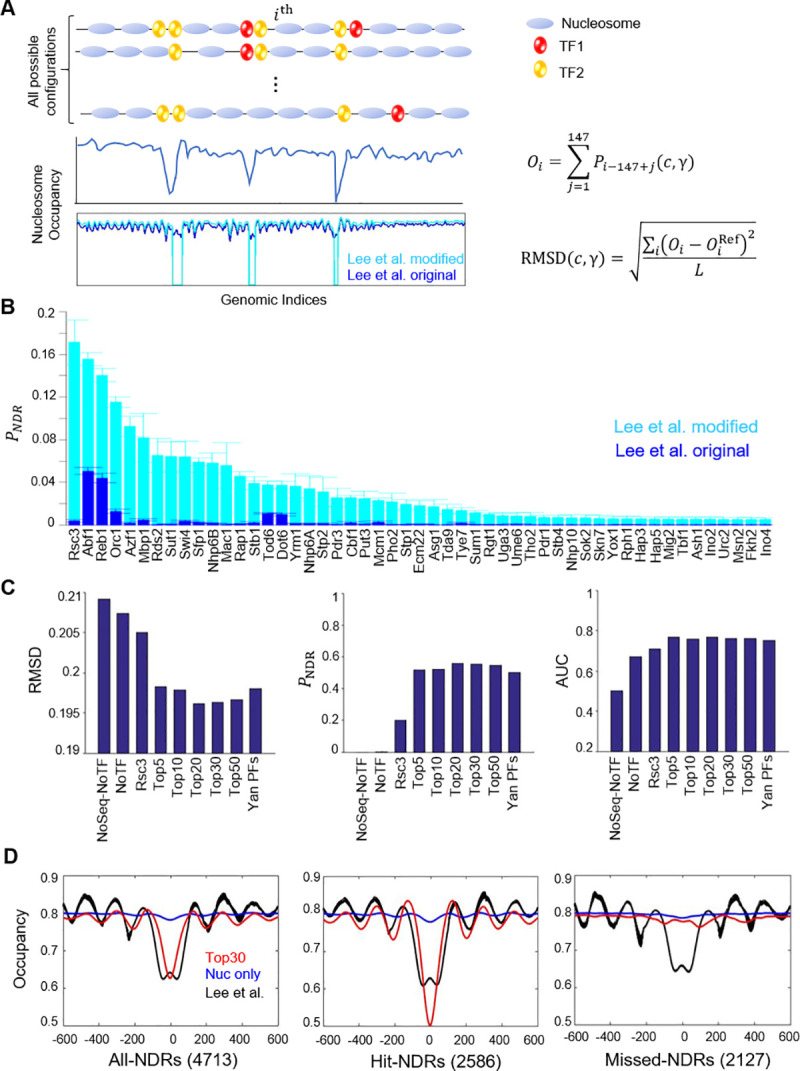
A thermodynamic model of TF-chromatin association to predict nucleosome positioning. **A)** Model construct. We defined “chromatin states” consisting of TFs (e.g., TF1, TF2) and nucleosome with different positioning patterns, and the occurrence of each state was determined by the total binding energy, a function of the concentrations (*c*) and binding specificities (γ) of histones and TFs. The occupancy of nucleosome (*O*_*i*_) at the *i*^*th*^ base pair was calculated by summing all the 147 overlapping Boltzmann weight factors (*P*_*i*_), where *P*_*i*_ is the probability of state with a nucleosome starting at the *i*^*th*^ base pair (see Materials and Methods). The best model parameters (*c* and γ) were obtained by minimizing the RMSD between the model and the experimental data. Two types of reference data were considered—original data from Lee et al. or modified (see main text). **B)** Prediction of NDR by incorporating individual TF into the model and optimizing the free parameters (“*c”* and “γ”) against the original or modified Lee et al. data. The TFs were ranked according to the fraction of NDRs that can be explained by the model (*P*_NDR_). **C)** The performance of models with different TFs taken into consideration. “NoSeq-NoTF” is a control that incorporates neither the intrinsic histone preference nor TFs (no sequence effect at all). The performance was evaluated by RMSD (root mean square deviation from the modified Lee et al. data), *P*_NDR_ (fraction of predicted NDRs), and AUC (area under a receiver operating characteristic curve). **D)** Average nucleosome occupancy aligned by the centers of all-NDRs, hit-NDRs, and missed-NDRs.

We started with a nucleosome positioning model considering the competitive binding of histones and TFs (**Fig 1A**; for details see Materials and Methods). Similar to the model in [26], we enumerated all possible configurations of nucleosomes and TFs with the requirement that their binding sites do not overlap. For each configuration, we calculated the binding energies of the nucleosomes based on the Segal lab model [3,15] and that of TFs based on their position weight matrices (PWMs) [27]. TF binding energies are only considered when the motif scores pass the recommended cutoffs [27]. These energy terms involve the concentrations and the binding specificities of histones and TFs (*c* and γ respectively), which are the tuning parameters in our model. The probability of each configuration was computed based on its total free energy using the Boltzmann factor, and the weighted average of these configurations yielded the nucleosome occupancy. Finally, we optimized the free parameters by minimizing the objective function of the difference between the computed and experimental nucleosome occupancy. To reduce the computational burden as well as to check for overfitting, we employed the 70/30 cross validation strategy, where 70% of the genomic data were used for optimization and the remaining 30% to crosscheck the fitted models.

Several genome-wide nucleosome measurements in *S*. *cerevisiae* have been published [3,5,6,28–31]. There are variations among these datasets, but all of them show nucleosome depletion near the transcription start sites (TSSs) and transcription termination sites (TTSs) (**S1 Fig**). In this work, we chose the measurement in Lee et al. to tune our model [6]. When fitted with the original nucleosome occupancy data in [6], our predictability of NDRs was poor even with optimized model parameters (**Fig 1B**). We hypothesized that this was because the model tried to fit the intermediate occupancy values in the NDRs, which likely result from low-level protection against micrococcal nuclease by non-histone proteins and do not reflect real nucleosome occupancy [32,33]. Indeed, since we started our project, a new nucleosome map generated by an alternative method showed much lower occupancy values in the NDRs [31]. We therefore modified the data in [6] by forcing the nucleosome occupancy in NDRs to zero (**Fig 1A**; also see Materials and Methods and **S2 Fig**). The model fitted with the “Lee-modified” data produced much higher NDR predictability (**Fig 1B**), and these modified data were used for further model fitting.

We first set up the model without any TFs and calculated the percentage of NDRs that can be predicted (*P*_NDR_). The value of *P*_NDR_ is highly sensitive to the NDR cutoff, and we chose a stringent cutoff of 0.6643 (two standard deviations away from the mean in the Lee et al. data) to reduce the false positive rate. To maintain the average nucleosome occupancy around 80%, close to that measured *in vivo*, we found that this model can only account for a very small fraction of genome-wide NDRs (**Fig 1C**). This result indicates that, at near-saturating histone concentration, nucleosome can form on almost any sequences, and the intrinsic histone binding preference is not sufficient in generating NDRs.

We next evaluated the effect of TFs on nucleosome positioning by incorporating TF one at a time into the model. In total, we tested 104 TFs that are present inside nucleus in the condition where the nucleosome occupancy was measured [6]. Ranking the TFs based on their contributions to *P*_NDR_ shows that well-known PFs, including Abf1, Reb1, Rap1, and Cbf1, are among the top contributors to the NDR prediction (**Fig 1B**). We then gradually increased the number of TFs in the model starting from the top contributor. In **Fig 1C**, we presented three quantities that describe the overall qualities of these models. The root-mean-square deviation (RMSD) between the model and the experimental data, which serves as the objective function, decreased sharply with the top five TFs and only showed minor improvement with the incorporation of the top 30 TFs. *P*_NDR_ and AUC (area under the ROC curve) showed the same trend as RMSD. These results indicate that only a few TFs significantly enhance the prediction of NDRs, which is consistent with the conclusion in Ozonov et al. [26]. This is also in agreement with our recent experimental study that only a subset of TFs (29 out 104) can function as PFs and open chromatin [16]. Interestingly, 15 out of the top 30 contributors, including all of the top five, belong to these 29 PFs [16]. An alternative model incorporating the 29 PFs showed slightly less but overall comparable predictability (**Fig 1C**). The reason that the NDR contributors in the model are not identical to the PF list in [16] will be explained in the discussion section. Overall, these data demonstrate that an equilibrium model considering the competitive binding of nucleosomes and a few PFs can largely account for nucleosome positioning pattern *in vivo*.

### Incorporation of TF cooperativity has little effect on NDR predictability

*P*_NDR_ and AUC reach the saturation levels of ~ 55% and ~ 75% in our model above (**Fig 1C**). Near the NDRs that are successfully predicted by the model (“hit-NDRs”), the simulated nucleosome occupancy agrees with the experimental data (**Fig 1D**). However, the model fails to predict any decrease in the nucleosome occupancy at the missed-NDRs (**Fig 1D**). To further improve the model performance, we first considered the possibility that the missed-NDRs are generated by the cooperative binding of multiple TFs. Numerous examples in literature show that adjacent TFs can enhance the binding of each other through attractive interactions, and TF clusters occur frequently in eukaryotic promoters and enhancers [34,35]. A recent study revealed extensive TF-TF interactions with distance between motifs up to 12 bp [34]. We reasoned that such cooperativity could stabilize weakly-bound TFs and allow them to compete more effectively with nucleosomes, leading to increased number of NDRs in the prediction.

**Fig 2:**
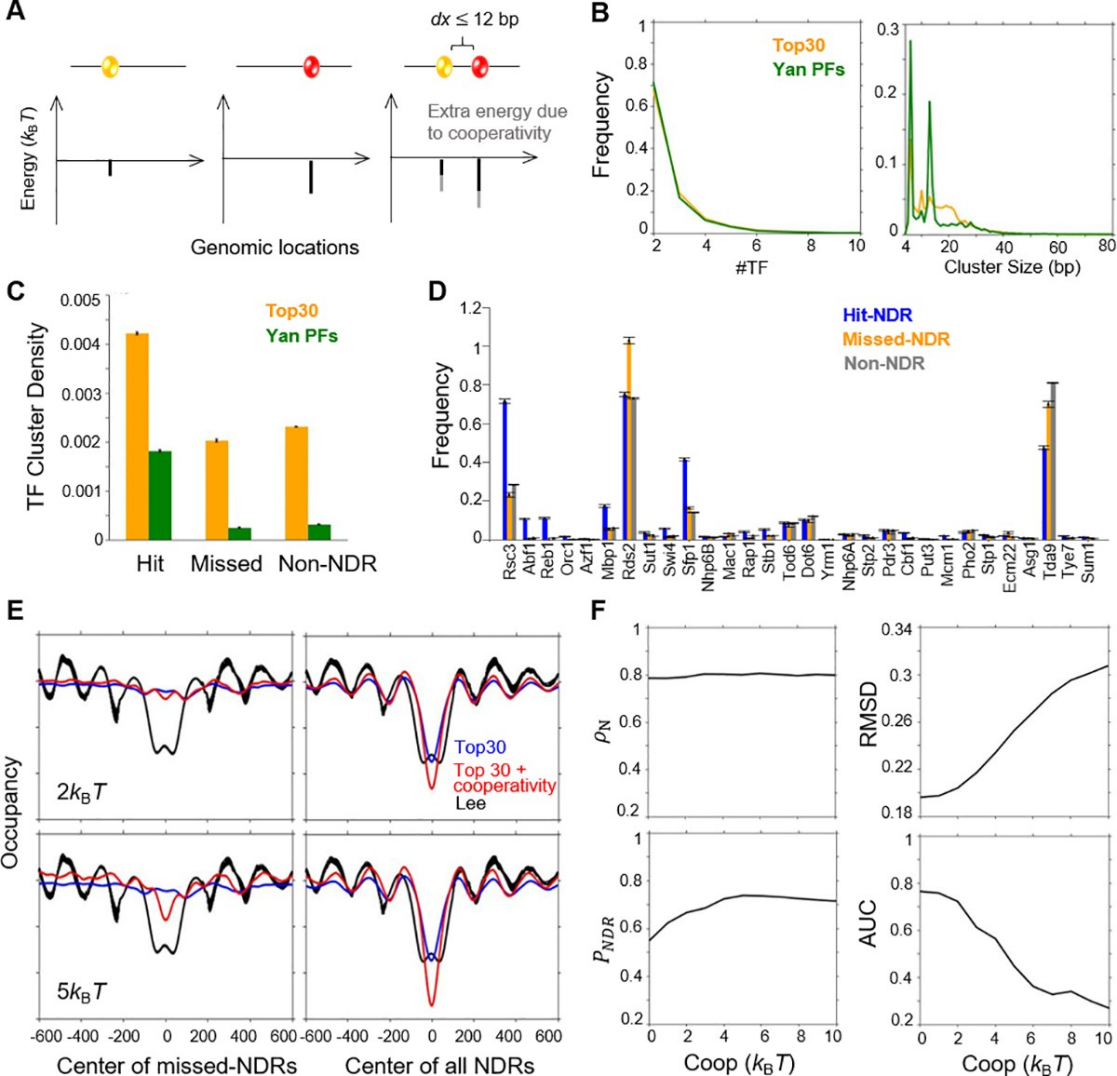
Incorporation of TF cooperativity has little effect on NDR predictability. **A)** Incorporation of TF cooperativity in the model. For two TFs separated by no more than 12bp, an extra TF binding energy (cooperativity strength) was introduced to both TFs. **B)** Distributions of number of TF within a cluster and size of TF clusters. **C)** TF cluster enrichments in hit-, missed-, and non-NDRs. **D)** Occurrence frequency of Top30 TF motifs in hit-, missed-, and non-NDR. **E)** Average nucleosome occupancy centered at missed-NDRs (left) and at all-NDRs (right) with cooperativity strength of 2*k*_B_*T* (top) and 5*k*_B_*T* (bottom). **F)** The performance of the model—*ρ*_N_ (average nucleosome occupancy), RMSD, *P*_NDR_, and AUC—as a function of cooperativity strength.

We introduced cooperativity into the model by identifying TF clusters in the genome and adding a constant energy term to each of the TFs in the cluster as the “cooperativity strength” (**Fig 2A**) (Materials and Methods). Non-overlapping TFs with distance in between their binding sites less than 12 bp (excluding the length of the motifs) are considered as a cluster. Clusters were found throughout the genome with on average ~ 3 TFs per cluster and a cluster size of ~ 16 bp (distance between the two TFs on the edges) (**Fig 2B**). Such TF clusters are highly enriched in hit-NDRs, and the consideration of the cooperativity can further strengthen the nucleosome depletion over these regions. However, both the density and composition of clusters are very similar between the missed-NDRs and the control nucleosomal regions (“non-NDRs”) (**Fig 2C and 2D**). The similarity between these two regions were unaffected even when the PFs found in Yan et al., more TFs, or larger cluster range were used in the calculation (**Figs 2C and S3**).

The observations in **Fig 2C and 2D** indicate that the cooperativity between the clustered TFs will not selectively enhance our ability to predict the missed-NDRs. Indeed, with high cooperativity strength (e.g. 5 *k*_B_*T*), the model predicts a fraction of the missed-NDRs (**Fig 2E**), but also generates many false positives. As a result, the overall model performance decreases significantly under these conditions (**Fig 2F**). Smaller values of cooperativity mildly enhance *P*_NDR_ without compromising RMSD and AUC. Overall, universal cooperativity among TFs has little effect on the NDR predictability, and therefore is not considered through the rest of this study.

### Alternative RSC binding mode significantly enhances NDR predictability

**Fig 3:**
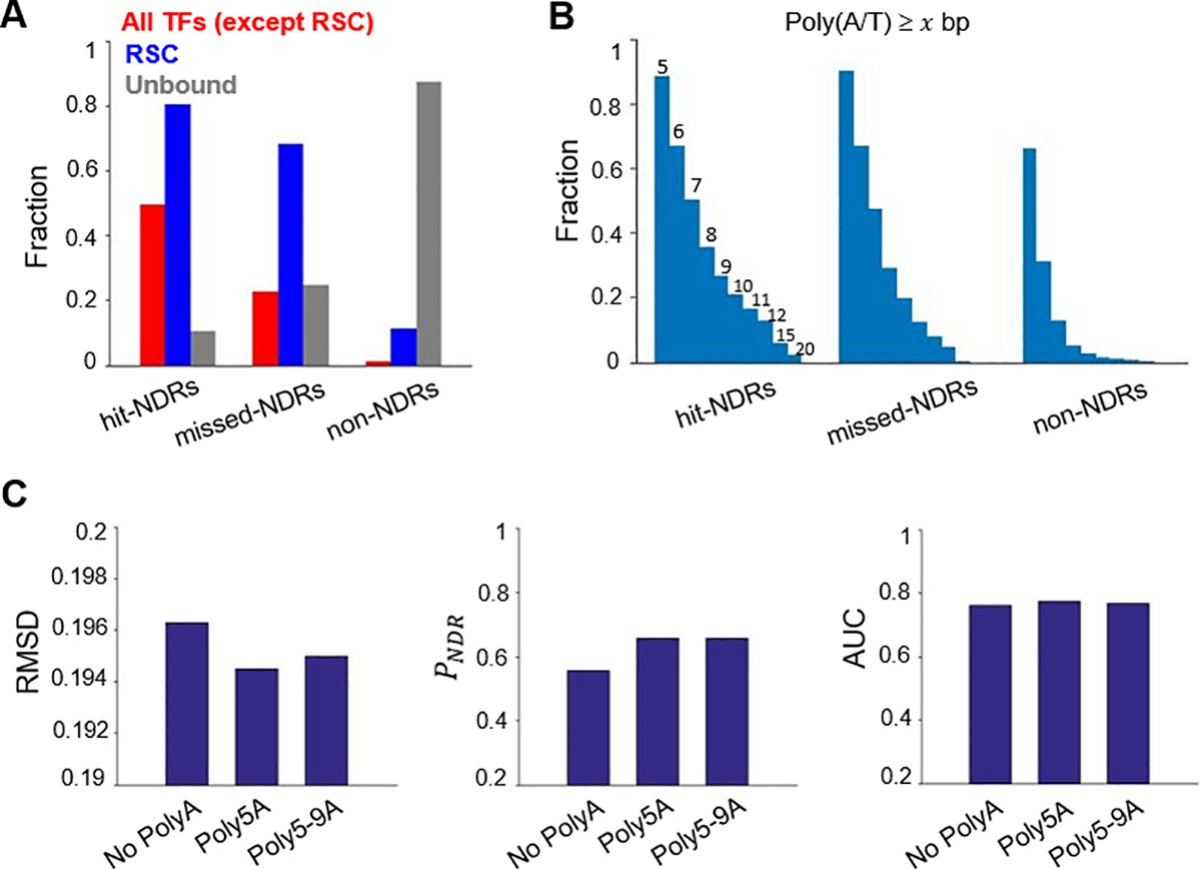
Alternative RSC binding mechanism significantly enhances NDR predictability. **A)** Fraction of hit-, missed-, and non-NDRs that show enrichment of TFs or RSC ChIP signals. **B)** Enrichment of Poly(A/T) tracks of different sizes in hit-, missed-, and non-NDRs. **C)** The performance of the model—RMSD, *P*_NDR_, and AUC—with no polyA (Top30 TFs only), polyA5 (Top30 TFs and polyA motif of length 5), and polyA5-9 (Top30 TFs and polyA motifs of lengths between 5 and 9).

Since the motif analysis above did not detect any major differences between the missed-NDRs and the non-NDR controls, we suspected that the motifs used here are not adequate in capturing the binding of some TFs. We therefore turned to the published ChIP-seq data [36–39] to identify TFs that are associated with the missed NDRs. Interestingly, the probability of finding ChIP peaks of sequence-specific TFs is indeed low in the missed-NDRs (**Fig 3A**). A remodeling complex, RSC, however, binds to a large fraction of the hit- and missed-NDRs, but not the non-NDR controls (**Fig 3A**).

RSC is known to associate with DNA through two distinct mechanisms. Two DNA-binding subunits of the RSC complex, Rsc3 and Rsc30, were shown to recognize “CGCGCG” motifs [40], which were already included in the model. RSC activity is also stimulated by the presence of poly(A/T) sequence in a Rsc3/30-independent manner [39,41,42]. Poly(A/T) tracks occur more frequently in the missed-NDRs than the non-NDRs (**Fig 3B**), consistent with the possibility that these sequences recruit RSC to deplete the local nucleosomes. We incorporated poly(A/T) motifs with various lengths into the model and evaluated the effect on the NDR prediction (Materials and Methods). Adding poly(A/T) motifs of 5 bp alone or of 5–9 bp significantly improved the model performance (**Fig 3C**). Together with the top 30 motifs, the *P*_NDR_ reaches ~ 66%. These results indicate that, instead of directly forming NDR through low intrinsic nucleosome stability, poly(A/T) tracks mediate nucleosome depletion through the action of RSC. Note that previous biochemical experiments led to the same conclusion [42].

### Consideration of remodeling activity allows more accurate prediction of the NDR sizes

**Fig 4:**
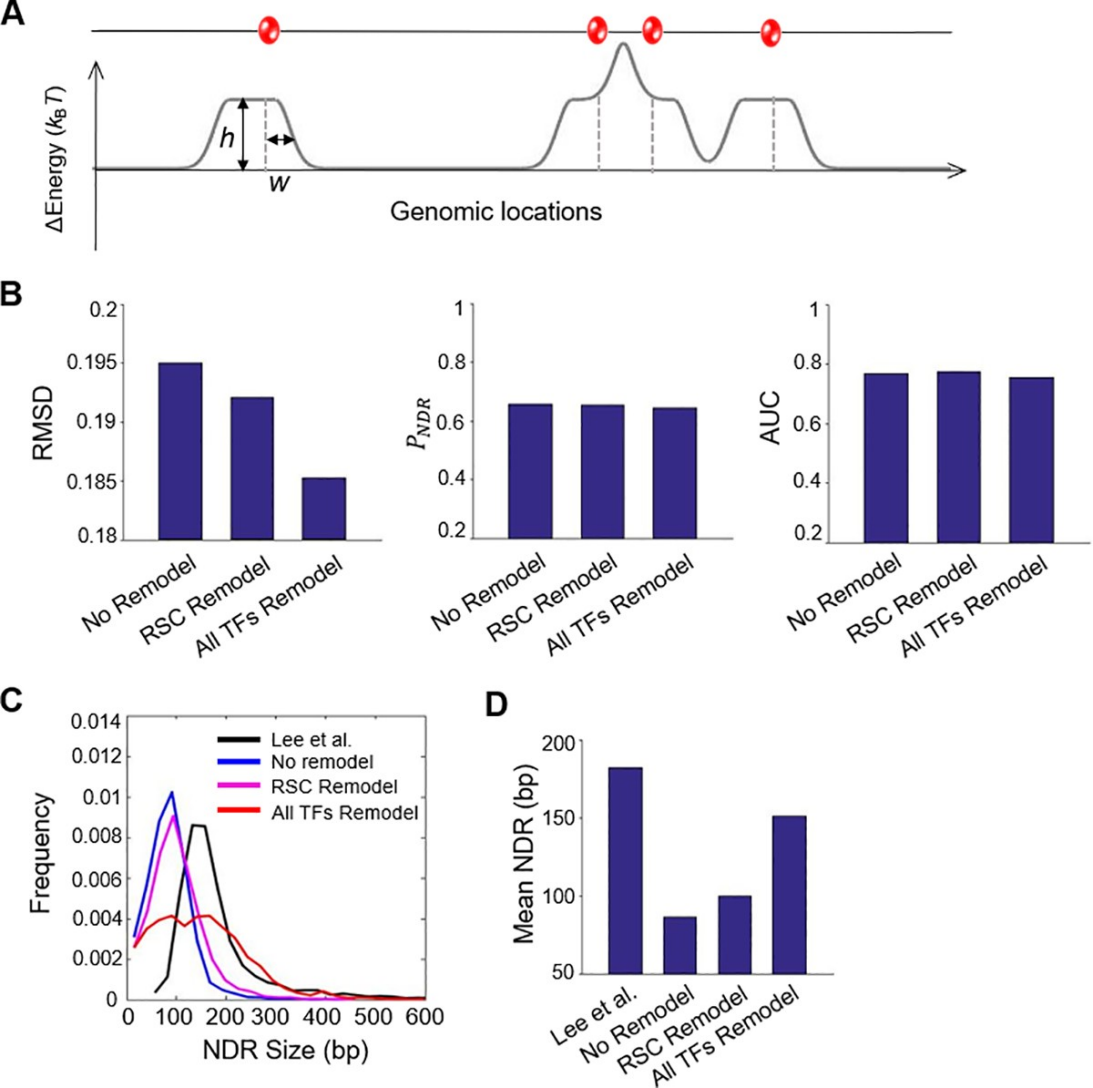
Consideration of remodeling activity more accurately predicts the NDR size. **A)** Modified energy landscape that incorporates the remodeling effect. A “soft” Gaussian potential is added adjacent to RSC sites (RSC remodel) or to all TFs (all TFs remodel) (see Material and Methods). **B)** Model performance with or without remodeling evaluated by RMSD, *P*_NDR_, and AUC. **C&D)** NDR size distribution (C) and average NDR length (D) in Lee et al. in comparison to different models.

In the model above, we treated all the TFs and RSC as physical blocks spanning the size of its recognition motif. Such treatment is obviously inadequate for RSC, given its activity of translocating on DNA and remodeling nucleosomes over a distance [38,43]. For other TFs, their footprints on DNA are also likely to exceed the range of their motifs. Moreover, they may coordinate with remodeling complexes to reposition the nearby nucleosomes and alter the NDR sizes. To take these effects into consideration, we modified the energy landscape by adding a Gaussian function with height *h* and width *w* in the vicinity of RSC or RSC + top 30 TFs (**Fig 4A**) [44,45]. The “*h*” is assumed to be proportional to the occupancy of the factor, and it can be either positive or negative, representing scenarios where the remodeler repels or attracts nucleosomes. The “*w”* is related to the working distance of the remodeler. In the RSC-only case, we modified the energy landscape near both the GC-rich and poly(A/T) motifs; in the RSC + top 30 TFs case, for simplicity, we assumed that all TFs and RSC have the same remodeling parameters.

Incorporation of the remodeling activity reduces the RMSD between the model simulation and experimental data (**Fig 4B**). The model with remodeling at RSC + top 30 TFs outperforms the RSC-only model, indicating that remodeling broadly exists over NDRs generated by many factors (see discussions below). The best fit was achieved for *h* ~ 16 *k*_B_*T* and *w* ~ 57 bp (see Materials and Methods). The positive energy shows that, on average, TFs push nucleosomes away from their binding sites. The width of the energy barrier, 57 bp, is significantly larger than typical TF footprint size (e.g. the footprint of Abf1 and Reb1 are 20–30 bp [46]), suggesting that the barrier is not due to the physical blockage of TFs, but requires remodeling activities. The improvement of RMSD is accomplished by a better prediction of the NDR sizes. Without remodeling, the mean size of NDRs is ~ 80 bp, much smaller than the measured length of ~ 180 bp (**Fig 4C and 4D**). With remodeling, the average size of NDRs becomes larger (~ 150 bp) and comparable to the experimental data, albeit with a broader distribution (**Fig 4C and 4D**).

### NDR formation over well-studied promoter regions

**Fig 5:**
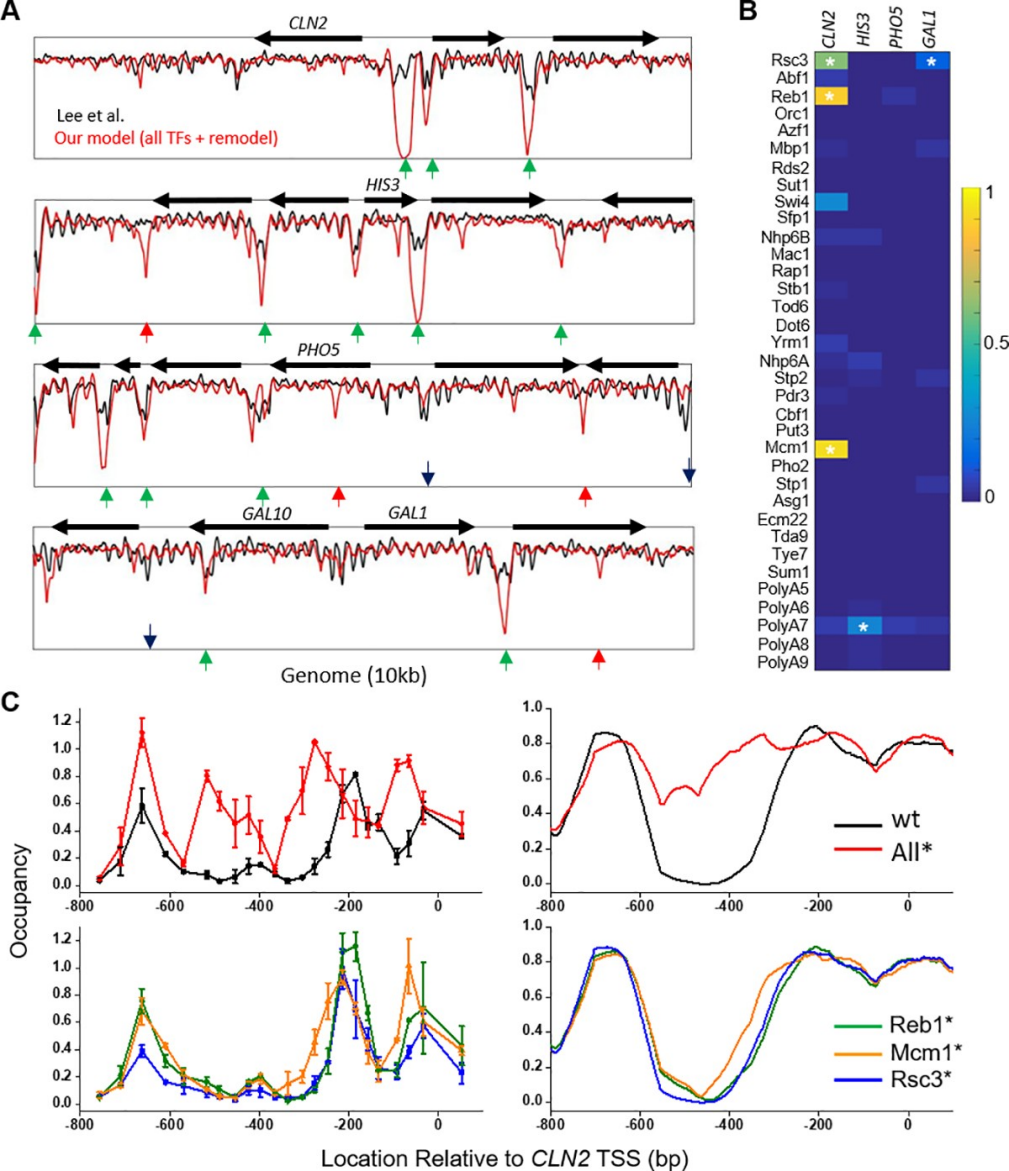
NDR formation over well-studied promoter regions. **A)** Nucleosome occupancy near the TSSs of *CLN2*, *HIS3*, *PHO5*, and *GAL1-10* genes. Green arrows: hit NDRs; black arrows: missed NDRs; red arrows: falsely predicted NDRs. **B)** Heatmap of the predicted TF occupancy (from 0–1) at the *CLN2*, *HIS3*, *PHO5*, and *GAL1-10* promoters. The highlighted TFs (*) are the ones that contribute to the formation of NDRs. **C)** Nucleosome occupancy at different *CLN2* promoter variants. Left two panels are experimental data, and the right ones are the corresponding simulation. wt: wild-type *CLN2* promoter; All*: simultaneous mutation in Reb1, Mcm1, and Rsc3 binding sites; Reb1*, Mcm1*, and Rsc3*: mutation in individual TF motifs.

**Fig 5A** shows the measured and predicted nucleosome occupancy over four well-studied genomic regions near the promoters of *CLN2*, *GAL1-10*, *HIS3*, and *PHO5*. Overall, the model predictions agree well with the experimental data. The factors that are predicted to generate the NDRs in the *CLN2*, *GAL1-10*, and *HIS3* promoters are highlighted in **Fig 5B**. Consistent with previous studies [38,47], Rsc3 sites and poly(A/T) have major roles in shaping the NDRs on the *GAL1-10* and *HIS3* promoters. Consistent with [48], multiple TFs, including Reb1, Mcm1, and Rsc3, contribute to the *CLN2* NDR formation. Genetic dissection experiment in the *CLN2* promoter has revealed that these factors work redundantly: mutations of Reb1 or Rsc3 sites have little effect on the NDR, mutation of the Mcm1 site mildly shrinks the NDR, and simultaneous mutations of all sites eliminate the NDR [48]. These results can be well recapitulated by the model (**Fig 5C**). The occupancies of NDR-generating TFs near transcription start sites, termination sites, and within gene bodies of all genes are listed in the supplementary tables (**S1, S2, and S3 Tables**) and visualized as heatmaps in **S4 Fig**.

### Further test of the model with independent datasets

To evaluate the model performance stringently, we compared the model prediction with experimental data that were not used for parameter tuning. For this purpose, we retrieved two published datasets—nucleosome occupancy on the native yeast genome upon factor depletion or on artificially introduced foreign DNA—to compare with our model prediction.

**Fig 6:**
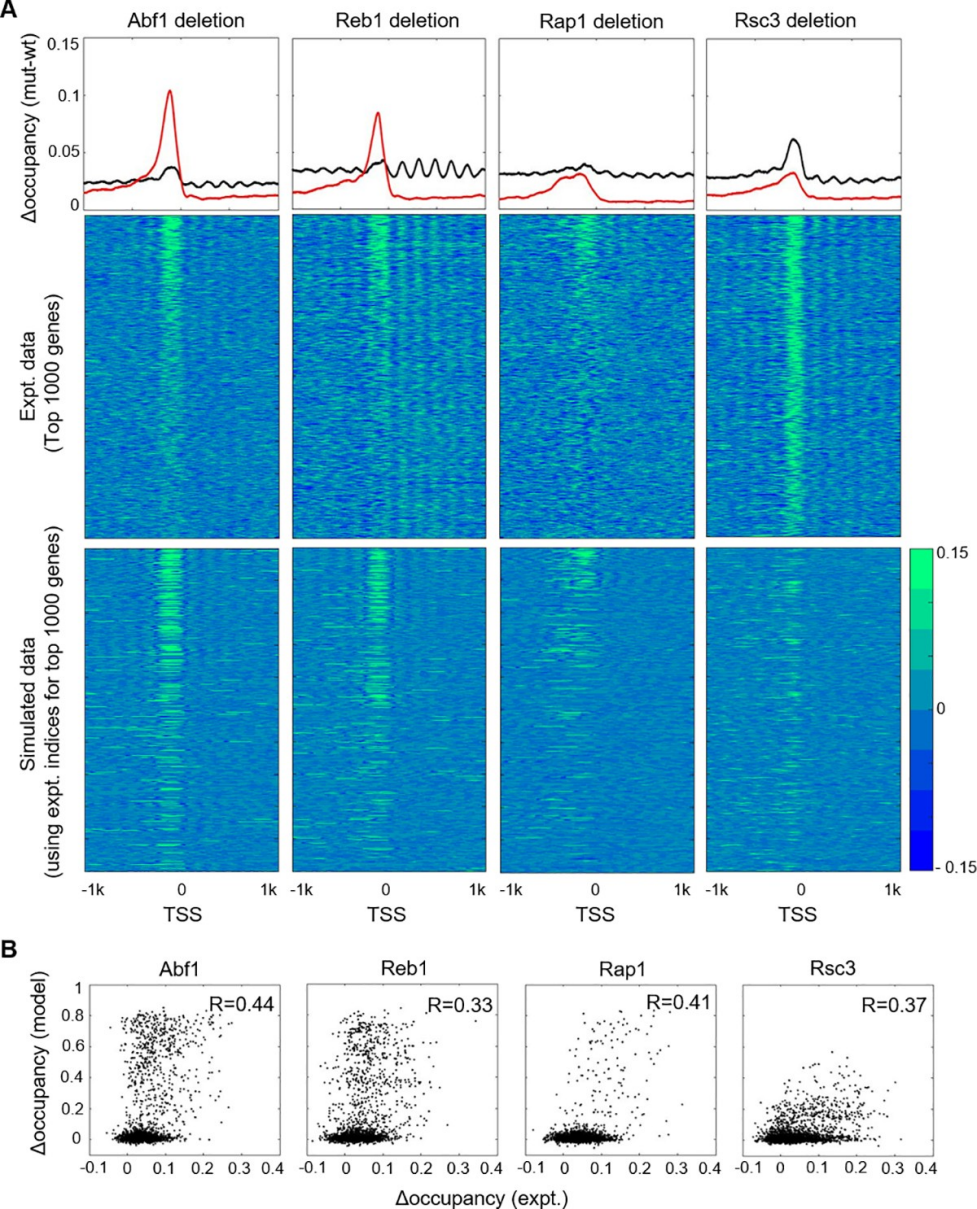
Change in nucleosome occupancy at TSSs upon TF deletion. **A)** Top row: composite plots of genome-wide nucleosome occupancy change near the TSSs upon the deletion of Abf1, Reb1, Rap1, and Rsc3. Our model (red) is compared with experimental (black) data. 2^nd^ and 3^rd^ rows: experimental and simulated heatmaps of nucleosome occupancy change. One thousand genes that show the largest occupancy changes in the experimental data are shown. **B)** Pearson correlation coefficient (R) between experimental versus simulated nucleosome occupancy change.

A previous study measured genome-wide nucleosome occupancy upon deletion of certain TFs [49]. We therefore eliminated these TFs in the model and compared the prediction of nucleosome occupancy with the experimental data. As expected, deletion of TFs that contribute to NDRs, including Abf1, Reb1, Rap1, and Rsc3, leads to increased nucleosome occupancy near TSSs (**Fig 6A**). The absolute value of the occupancy change tends to be higher in the model, largely because the model was tuned based on the modified Lee et al. data (**Fig 1A**) and predicts lower occupancies in the NDRs in the presence of these TFs. Nevertheless, a gene-by-gene comparison shows that the predicted occupancy change is well-correlated with the experimental data (**Fig 6A and 6B**). The Rsc3 deletion data is better predicted by the model that eliminates the contribution from the Rsc3 motif than the one that eliminates both the Rsc3 and poly(A/T) motifs or all remodeling activities (**S5 Fig**), consistent with the previous finding that RSC binding on poly(A/T) sequences is independent of the Rsc3. Overall, these data show that our model can partially reflect the nucleosome occupancy change upon TF mutations.

**Fig 7:**
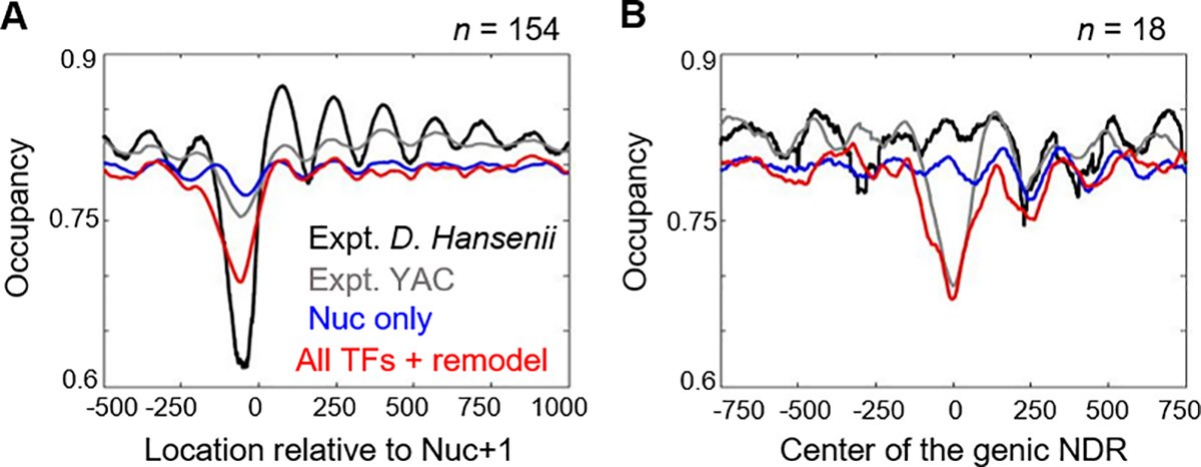
Nucleosome occupancy on foreign DNA. Experimental and simulated nucleosome occupancy near the +1 nucleosome **(A)** and fortuitous NDRs inside gene body **(B)**. The black and grey curves represent the measured nucleosome occupancy in *D*. *Hansenii* (endogenous) and *S*. *cerevisiae* (YAC) respectively. The blue and red curves represent simulated data with or without TF and remodeling.

We next compared our model predictions with another nucleosome dataset measured on the *D*. *Hansenii*-derived yeast artificial chromosomes (YACs) introduced into *S*. *cerevisiae* [50,51]. For the 154 promoters in the YACs, a fraction of their NDRs is maintained from *D*. *Hansenii* to *S*. *cerevisiae*. In addition, new NDRs appeared in the coding regions in the YACs, which were proposed to be generated by fortuitous binding of certain *S*. *cerevisiae* TFs [50]. Both types of NDRs can be well-reproduced by our model (**Fig 7**). Interestingly, the model shows that Abf1, Reb1, and PolyA7 are responsible to generate the *D*. *Hansenii* coding-region NDRs in *S*. *cerevisiae* (**S6 Fig**). *D*. *Hansenii* has no Abf1 orthologue, and its Reb1 has alternative functions [52], which readily explain why these NDRs show up in *S*. *cerevisiae* but are absent in the native environment. The presence of PolyA7 in these NDRs indicates that RSC in *D*. *hansenii* may have less affinity to the poly(A/T) sequences. Indeed, PolyA7 was found to be less nucleosome-depleted in *D*. *hansenii* than other fungal species [52]. This example shows that our model can be used to predict nucleosome positioning on novel DNA and generate insights into the NDR formation mechanism in different species.

### Comparison with other related models

**Fig 8:**
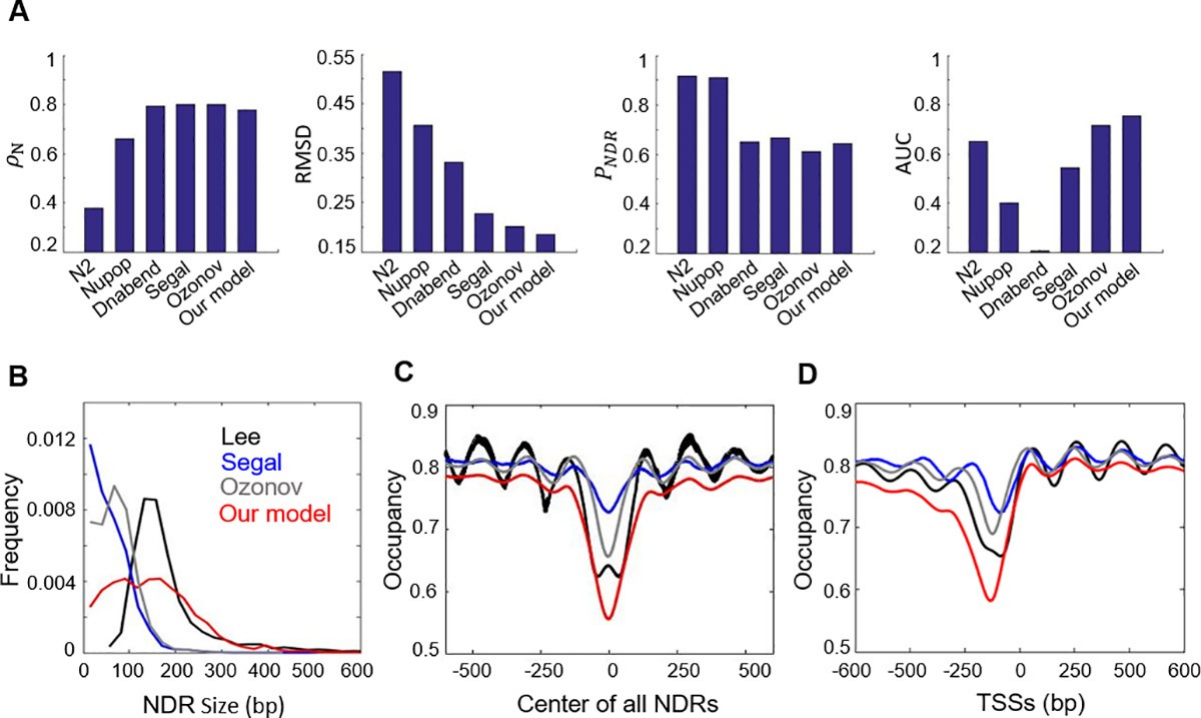
Comparison with other related models. **A)** Model performance measured by *ρ*_N_, RMSD, *P*_NDR_, and AUC are compared between different models: N2, Nupop, Dnabend, Segal, Ozonov, and our own model. Here, the RMSD, *P*_NDR_, and AUC were calculated by using Lee et al. datasets as the reference datasets. **B)** Distribution of NDR sizes measured in Lee et al. in comparison to the ones predicted by the indicated models. **C)** Nucleosome occupancy at the centers of all the reference NDRs (**S4 Table**). **D)** Nucleosome occupancy relative to the TSSs for all the genes (TSSs annotations adapted from Chereji et al. [44]).

Many nucleosome positioning models have been proposed. The models vary from solely based on the histone affinities [15,24], to considering the frequency of short DNA sequences (some of which may reflect TF motifs indirectly) [53], to explicitly incorporating the TF contributions [26,54]. In **Fig 8A**, we compared the performance of our model with some of these models. Bars 1–4 represent models without explicit consideration of TFs [15,24,25,53], and 5 is from the model in Ozonov et al. [26]. The models with TFs performed significantly better as indicated by RMSD and AUC. More specifically, the models without the TFs, especially N2 [53] and Nupop [25], require low average nucleosome density (*ρ*_N_) to achieve high *P*_NDR_, which leads to high false-positive rate and low AUC. Dnabend [24] and Segal [15] can provide ~ 80% average occupancy, but both produce low AUC: Dnabend and Segal have AUC ~ 0.2 and ~ 0.55 respectively. The model in Ozonov et al. [26] has similar performance as our model in **Fig 1** (with Top 30 TFs but no considerations of the RSC-poly(A/T) interaction and remodeling), and we have shown that these extra considerations improve the NDR prediction (**Figs 3 and 4**). To avoid bias in the comparison due to our modification of the Lee et al. data, we also compared different models using the original measurements in Oberbeckmann et al. [31]. The results are similar to Fig 8A, and our model still outperforms other models in terms of RMSD and AUC (**S7 Fig**). Most of these models generate NDRs of very narrow sizes, and remodeling activity is required to expand the NDRs to the measured level (**Fig 8B**). In addition, the degree of nucleosome depletion over the NDRs and the positioning of neighboring nucleosomes can be better recapitulated in our model (**Fig 8C and 8D)**. Overall, our model significantly outperforms these previous models and represents major improvement of the predictability of nucleosome positioning in yeast.

## Discussion

In this study, we built a thermodynamic model to compute the nucleosome occupancy by averaging over all possible conformations defined by TF and histone binding energies. In our model, histone binding specificity is relatively weak (*γ* ≈ 0.15–0.2), and by itself, unable to direct NDR formation. Such low specificity is consistent with previous findings of *γ* ≈ 0.1–0.5 [26]. Instead, incorporation of a small fraction of TFs is both necessary and sufficient to predict the location of most NDRs. Among these factors, the remodeling complex RSC, through its two modes of sequence-specific DNA binding, is the most significant contributor of NDRs. In one of these modes, RSC recognizes poly(A/T) sequences, which also directly reduces the histone affinity. Consistent with previous experimental data [39,42], our model quantitatively proves that poly(A/T) mainly functions through RSC to generate NDRs, not through the intrinsic stability.

The model performance reaches a saturation level when the top 30 TF contributors are incorporated. A recent study screened genome-wide yeast TFs and found 29 PFs that have nucleosome-displacing activities [16], including 6 strong and 23 weak PFs (displace nucleosome with a single or multiple motif, respectively). All the strong PFs and 9 of the weak PFs are included in the top 30 contributors. Since nucleosome depletion by the weak PFs requires multiple closely-spaced motifs, which may have small number of occurrences in the native genome, it is not surprising that some of these PFs do not have strong impact on the genome-wide nucleosome positioning. On the other hand, the top 30 contributors also include TFs that do not seem to have nucleosome-displacing activities, such as Mbp1 and Swi4 [16]. One possible explanation is that, even though these factors cannot directly invade into nucleosome-covered regions, they can maintain or even expand previously existing NDRs. In fact, once Swi4 binds to the *HO* promoter, it can recruit FACT, SAGA, and SWI/SNF to deplete the neighboring nucleosomes [55]. Incorporation of this type of factors, therefore, may enhance the predictability of NDRs.

To improve the model performance, we found it necessary to artificially set the nucleosome occupancy in NDRs to zero (**Fig 1B**). This treatment is consistent with previous findings that NDRs in the promoters are largely depleted of histones, and the protection seen with low MNase is likely due to non-histone complexes [33]. In addition, in previous low-throughput measurements, nucleosome occupancy over NDR regions are very low (often less than 0.2), and transcription driven by these promoter NDRs are homogenous among cell population [7,16,48], supporting the notion that NDRs are mostly nucleosome-free. Finally, recent nucleosome mapping in yeast using restriction enzymes and DNA methyltransferases [31] shows very low nucleosome occupancy in NDRs, and our model prediction is consistent with these data without the need to artificially lowering the nucleosome occupancy (**S7 and S8 Figs**).

Our model also considers the effects of TF cooperativity and remodeling. Due to the lack of factor-specific information, we took an over-simplistic approach of assuming that all the top 30 TFs have the same cooperative binding. Such cooperativity treatment did not significantly improve the prediction of nucleosome positioning. These results may indicate that cooperativity is not a general property of yeast TFs, and most NDRs are generated by TFs independently, or even redundantly. This is consistent with previous analysis that only 0.1–0.2% of yeast TF pairs show cooperative binding [56]. Besides direct binding cooperativity, previous study also proposed a nucleosome-mediated cooperativity [57]. This model considers competition between multiple TFs with a single nucleosome that has fixed translational positioning, and the cooperativity comes from the release of a long stretch of DNA that is available for TF binding upon nucleosome depletion. In contrast, our model considers nucleosome arrays where nucleosomes can adopt different translational positioning to accommodate TF binding. Therefore, the two models cannot be directly compared. Nevertheless, our model also has intrinsic “nucleosome-mediated cooperativity” in the sense that adjacently bound TFs can be much more effective in displacing local nucleosomes than individual TFs by generating chromatin states with significantly lowered free energy.

The consideration of remodeling significantly improves the model. The optimized remodeling parameters suggest that, on average, TFs present high energy barriers to exclude nucleosomes in a ~ ±60 bp range. We speculate that most of these effects are generated by RSC, because the deletion of RSC, but not other remodeling factors, causes shrinkage of a large fraction of genome-wide NDRs [58]. Interestingly, a model with remodeling barriers at all TFs performs better than the one at RSC motifs only (GC-rich + poly(A/T)). This result indicates that, besides sequence-specific binding, RSC can be recruited to a broad range of NDRs either through interactions with certain TFs or non-specific interactions with naked DNA.

Our model fails to predict ~ 34% of the NDRs. Part of the reason here is related to the NDR annotation. Because of experimental variation and differences in criteria, past studies have designated different genomic regions as NDRs [44,59], which partially overlap with the NDRs used here (**S2 Fig**). Some of the missed NDRs thus may not be real. However, even for the NDRs that are common among all datasets, our predictions are still not perfect (the *P*_NDR_ is ~ 72%, **S8 Fig**), so there are real NDRs that are missed by the model. According to the published ChIP data, a large fraction of the missed NDRs is associated with RSC but not with other sequence-specific TFs (**Fig 3A**). Therefore, the deficiency in NDR prediction is related to the inaccuracy of the RSC binding motif. In the current model, we assume that RSC binds to a continuous stretch of poly(A/T), but poly(A/T) interrupted by other bases may also contribute to RSC binding. For example, “T_4_ACT_7_” sequence in the *PHO5* promoter and “T_3_GT_6_” in the *GAL2* promoter were found to associate with RSC and cause nucleosome depletion [42]. The affinity of RSC towards the short poly(A/T) stretches in these sequences (T_4_ and T_3_) is not considered in the current model. As a result, our model predicts weak RSC binding and nucleosome depletion on these promoters (**Fig 5A**). We believe that more quantitative measurement of RSC affinity on various A/T rich sequences will improve the NDR prediction.

Besides false negative prediction of NDRs, our model also shows some false positives. Some TFs can apparently bind to chromatin and organize tightly packed nucleosome arrays, and these factors may explain why some NDRs predicted by our model are absent *in vivo*. To test this idea, we examined Tup1, a factor that was suggested to stabilize nucleosomes [60,61]. We collected Tup1-target genes (161 with verified ORF) using the YEASTRACT database [62], and found that about half of these genes (79) have false positive NDRs in their promoters (within 600bp upstream of the ORF). In contrast, when we did the same analysis on Reb1 and Abf1, we found false positive NDRs in ~ 25% of their target promoters. This result is consistent with the possibility that some TFs, including Tup1, may “close up” NDRs, and we plan to investigate this type of TFs more systematically in the future.

Another aspect of the model that requires further improvement is the NDR length prediction. After the incorporation of remodeling, the average NDR length predicted by the model is consistent with experiments (**Fig 4D**). However, the variation of the NDR length is significantly larger (**Fig 4C**). As a result, the predicted nucleosome positioning near NDRs shows less “phasing” in comparison to the experimental data (**Fig 8B and 8C**). One possible reason here is that the model only considers remodeling that leads to nucleosome exclusion. In the cells, the length of NDR results from a balancing act among nucleosome “pushers” and “pullers” that lead to NDR broadening or shrinkage [58]. It is possible that long NDRs will recruit more “pullers”, which serves as a negative feedback that limits the variation of NDR length. In addition, some remodelers may have specific interactions with certain TFs, which will also affect the NDR length distribution. The model will benefit from a future study that examine the relation between PFs, NDRs, and remodeling factors.

## Materials and methods

### A steric model for chromatin and calculation of partition functions

We used a statistical mechanics approach to study genome-wide nucleosome organization in the presence of TFs. Here, the DNA was treated as a long 1-D lattice where nucleosomes and TFs compete for genomic spaces (**Fig 1A**). The lattice constant of the DNA is 1 bp. The size of nucleosome is 147 bp and the sizes of TFs are given by length of their position weight matrices (PWMs). The particles (nucleosome and the TFs) are not allowed to overlap during simulation. For every chromatin configuration we assigned a statistical ‘weight’ proportional to the exponential of the binding energies of the particles also known as the Boltzmann factor. Sum of the weights of all these configurations is the partition function of the system. Thus, the probability of observing the *n*^*th*^ state is given by:

$$p_{n}=\frac{g_{n}}{Z}$$ (1)

where *g*_*n*_ is the statistical “weight” of the *n*^*th*^ state and *Z* is the partition function such that

$$Z=\sum_{n}g_{n}$$ (2)

Let us denote *G*(*i*) as the sum of all the weights of those states with particle *t* starting at the *i*^*th*^ position. Then, *P*_*t*_(*i*), the probability of finding the particle *t* starting at the *i*^*th*^ position, is given by:

$$P_{t}(i)=\frac{G(i)}{Z},$$ (3)

where *G*_*i*_ is given by:

$$G(i)=\sum_{n\in \{i\}}g_{n}.$$ (4)

Using *P*_*t*_, we calculated the occupancy of any particle at a given location using the formula:

$$O(i)=\sum_{j=i-l_{t}+1}^{i}P_{t}(j)$$ (5)

where *l*_*t*_ is the size of the particle *t* in unit of base pairs. The partition functions allowed us to get the required particle occupancies. One of the common methods used to calculate *Z* (and *G*_*i*_) is to express *Z* as forward and backward recursive functions [24,26,54]. Thus, the partition function (forward) is given by:

$$Z^{f}(i)=Z^{f}(i-1)+\sum_{t}Z^{f}\left(i-l_{t}\right)c_{t}e^{-\gamma_{t}E_{t}(i-l_{t}+1)}$$ (6)

where *Z*^f^({*l*_*t*_}_*min*_−1) = ⋯ = *Z*^f^(0) = 1.

Backward partition function:

$$Z^{b}(i)=Z^{b}(i+1)+\sum_{t}Z^{b}\left(i+l_{t}\right)c_{t}e^{-\gamma_{t}E_{t}(i)}$$ (7)

where *Z*^b^(*L*−{*l*_*t*_}_*min*_+2} = ⋯ = *Z*^b^(*L*+1) = 1, and *L* is the size of genome. It is easy to show that *Z*^f^(*L*) = *Z*^b^(*L*) = *Z*.

Using Eqs (6) and (7), Eq (3) can be rewritten as:

$$P_{t}(i)=\frac{Z^{f}\left(i-1\right)Z^{b}\left(i+l_{t}\right)c_{t}e^{-\gamma_{t}E_{t}(i)}}{Z}$$ (8)

where *E*_*t*_ is the binding energy of particle *t* on the DNA starting at the *i*^*th*^ position, and the scaling parameters, *c*_*t*_ and *γ*_*t*_, are the concentration and the binding specificity of the particle. Below we described how to obtain these three quantities: *E*, *c* and *γ*.

The binding energy of nucleosome was obtained using the nucleosome prediction software by Segal lab (http://genie.weizmann.ac.il/software/nucleo_prediction.html) with default parameters. The raw log-ratio of the binding probability can be written as the binding energy:

$$E(i)=-\log\;P_{N}(i)+E_{N}$$ (9)

where *P*_*N*_(*i*) is the binding probability of nucleosome starting at the *i*^*th*^ position and *E*_*N*_ is the sequence-independent energy term.

The binding energy of TFs was obtained by scanning the PWMs along the genome and converting into position-dependent energy:

$$E(i)=-\sum_{j=1}^{l}\log\left[w_{i}(j,\alpha_{j})\right]+E_{T},$$ (10)

where *w*_*i*_(*j*, *α*_*j*_) is the weight of *α* bp at *j*^*th*^ position within a TF of size *l* starting at *i*^*th*^ position on the DNA and *E*_*T*_ is the sequence independent energy term. By setting average binding probability as 〈*e*^−*E*(*i*)^〉 = 1, we obtain *E*_*N*_ or $E_{T}=\log\left(\langle \frac{1}{e^{E_{0}}}\rangle \right)$ where *E*_0_ is the first term on the right hand of either Eqs (9) or (10), and 〈 〉 is the average over the entire genome. The assumption of 〈*e*^−*E*(*i*)^〉 = 1 is not important, because any change in *E*_*N*_ or *E*_*T*_ can be compensated by the fitting parameter *c*_*n*_ or *c*_*t*_. The purpose here is that, by setting *E*_*T*_ this way, we provided scaling factors so that *c*_*t*_ for different TFs are not drastically different so that it is easier to find the best fit.

### Parameter fitting

The scaling parameters (*c*, *γ*) can be estimated by optimizing the degree of overlap in genome-wide nucleosome occupancy between our model, *O*, and the experimental data, *O*_*Lee*_ [6]. This is given by the root-mean-square deviation of all base pairs, *O*_*rmsd*_, between *O* and *O*_*Lee*_:

$$O_{rmsd}=\sqrt{\frac{\sum_{i}{\left(O(i)-O_{Lee}(i)\right)}^{2}}{L}}$$ (11)

As described in [26], the *in vivo* nucleosome occupancy data in [6] has a few exceptionally high values which we discarded so as not to skew the parameter fitting. We then obtained two variants of Lee datasets with (Lee-modified) or without (Lee-original) NDR modifications. For Lee-modified data, we set nucleosome occupancy to zero in the genomic regions that are annotated as NDRs. See below for a detailed algorithm for locating NDRs.

For the minimization of our objective function, *O*_*rmsd*_, with respect to (*c*, *γ*), we used the Nelder-Mead simplex algorithm in conjunction with simulated annealing [63]. To minimize the computational cost as well as to check for overfitting, we employed the 70/30 cross validation strategy. Here, 70% of the genomic data was used for optimization and the remaining 30% to crosscheck the fitted models. The parameter-fitting step was repeated five times and each time the chromosomes were shuffled randomly such that any two sets have different genome partitions. Finally, the parameter set that gave the best *P*_NDR_ was chosen as the best fit and used in computing the occupancies.

All 74 best-fit parameters (30x2 for TFs, two for nucleosome, 5x2 for PolyAs, and two additional remodeling parameters) used in the model are listed in **S4 Table**. All the coding and analyses were written using MATLAB R2016a and R2019a.

### Locating and annotating NDRs

The log-scale occupancy data (*Y*_L_) in Lee et al. was transformed into absolute occupancy so that it lies between 0 to 1 after skimming way data points *Y*_L_>1.26. The absolute occupancy is given by: $Y=ce^{\gamma Y_{L}}$. We obtained *c* = 0.8244 and *γ* = 0.1581 by setting the genome average, 〈*Y*〉 = 80% and confining *Y* within 0 to 1. We chose the highest possible value of *γ* to “stretch” the occupancy curve. In order to identify and locate NDR, the occupancy was discretized by horizontal lines with the starting line at 80%, 73.2%, 66.4%, etc. (**S2A Fig**). We assigned regions with occupancy < 80% as potential NDR candidates, from which we selected a subset based on the following criteria: 1) The width of the occupancy curve that crosses the 73.2% line (including the small “bump” in the middle) needs to be > 110 bp. 2) The drop of nucleosome occupancy in NDR needs to be relatively steep (the distance between the cross point with the 80% line and the lowest cross point need to be < 100 bp) (*dx* in **S2B and S2C Fig**). 3) If any two neighboring centers of NDR are separated by less than 125 bp, they are merged into one NDR. For the region between the lowest cross-points at the left and right ends of the NDR, we set the occupancy to 0. Finally, since the genome average of the modified occupancy is slightly less than 80%, we transformed *Y* back to log data and recalculate *c* and *γ* using Eq 1 to get new *Y* with 80% average occupancy. Using the modified map, the NDR length was defined as the length of the region that fall below 66.43%.

We found 4713 NDRs in total. The NDRs in one example region, as well as the comparison to previously annotated NDRs from other studies, are shown in **S2D Fig**. Our annotated NDRs consist mainly of larger size (>100 bp) so that they represent the probable targets for TFs. The smaller NDR sizes may represent normal linker DNA as a consequence of statistical positioning. The positions of NDR from our study share ~ 63% and ~ 62% match with Chereji et al. [44] and Yadon et al. [59] respectively (**S2E Fig**). We compared our model predictions with NDRs annotated from different studies (**S8 Fig**). With the exception of Yadon et al., which defined a larger set of NDRs, our model agrees to a similar extent to the NDRs in all the other studies. The coordinates of the NDRs found in Lee et al. and Oberbeckmann et al. are listed in **S5 and S6 Tables,** respectively.

### Cooperative binding of TFs

In order to identify TF clusters that can participate in cooperativity, we lump TF binding sites into clusters if the distance between two TFs is less than “*dx*” (we have tried both 12 and 147 bp). A cluster may consist of two or more TFs. When multiple TFs associate with overlapping motifs, we consider the top two motifs with highest scores and allow them to bind simultaneously, which can happen in some cases [34]. The cooperative binding is achieved by increasing the binding specificities of all the TFs in the cluster by an equal amount. Because the cooperative binding reduces the average nucleosome occupancy, we readjusted the *c* and *γ* of nucleosome when carrying out the optimization step.

### TF associated nucleosome remodeling activity

We incorporate the remodeling effect by modifying the binding energy of nucleosomes adjacent to bound TFs. We assume the modification to be a Gaussian function centered around the TFs as depicted in Fig 4A. We also do not consider secondary remodeling (remodeling generated by new TFs that are bound due to the first remodeling event). For the sake of simplicity, we assume that (*h*, *w*) is the same for all TFs. We also assume that the remodeling potentials for adjacent TFs can be superposed (**Fig 4A**). To fit *h* and *w*, we first used the fitted nucleosome and TFs parameters (*c*, *γ*) to obtain the occupancy of TFs. We consider all TFs with occupancy > 0.0022, and add the remodeling potential near the TF based on the occupancy. For example, if a TF occupancy is 10%, we simulate the energy landscape 100 times, and add the modification 10 times. This is carried out for all the bound TFs genome-wide. The final occupancy of the nucleosomes are given by the average of the 100 repeats. The optimal values of the remodeling parametrs (*h*, *w*) were obtained by carrying out the optimization step using (*c*, *γ*) of nucleosome and the (*h*, *w*) as variables while keeping (*c*, *γ*) of TFs as constants.

## Supporting information

S1 Fig Comparison of *in vivo* nucleosome occupancy in a few datasets. **A)** Histograms of nucleosome occupancy: Lee et al. [6], Kaplan et al. [3], Mavrich et al. [29], and Oberbeckmann et al. [31]. **B)** Heatmap of the root-mean-square-deviation (RMSD) between different datasets. **C)** Composite plot of the average nucleosome occupancy near transcription start sites (TSSs, left) or transcription termination sites (TTSs, right).
(PPTX)

S2 Fig Locating and annotating NDR. **A)** A snapshot of Lee et al. occupancy data [6] showing three examples of potential NDRs: (a), (b), and (c). The occupancy data is discretized using horizontal lines at 80%, 73.2%, 66.4%, etc. with a constant step size of 6.78%. **B)** Zoom-in views of the annotated NDRs, (a)–(c), in A. “*dx*” is the distance between a cut-point at 80% line and the lowest crossing points. “*l*” is the distance between cross points with the 73.2% line. The occupancy was modified so that the occupancies between the lowest cross points are set to 0. The size of NDR is indicated by the red lines (length crossed at the 66.4% line). **C)** A histogram of *dx*. For NDR annotation, we require *dx* to be less than 100bp. **D)** Modified occupancy in an example region with the sharp downward spikes to 0 occupancy representing NDRs. The original data is shown in blue. The different asterisk points represent locations of annotated NDRs from previous studies: Chereji et al. [44], Yadon et al. [59], and Jiang & Pugh [64]. **E)** A table showing the number of overlapping NDRs between our annotation and those from Chereji et al. and Yadon et al.
(PPTX)

S3 Fig TF cluster distribution, density, and composition. **A)** TF cluster density in hit-, missed-, and non-NDRs with different number of TFs taken into consideration (maximum distance between TFs in a cluster, *dx*, is 12bp). **B)** Histograms for the number of TFs in a cluster (left) and the size of TF clusters (right) with *dx* = 147bp. **C)** TF cluster density in hit-, missed-, and non-NDRs with *dx* = 147bp. **D)** Occurrence frequency for Top30 TF motifs in hit-, missed-, and non-NDRs.
(PPTX)

S4 Fig **Heatmap of the occupancy of the top 30 TFs and the five PolyA factors near TSSs (top), within gene bodies (center), and near TTSs (bottom).** We have listed all 5542 genes with the exact TSS and TTS coordinates, and corresponding values can be found in S1, S2 and S3 Tables. The gene indices and annotations are adapted from ref. [44].
(PPTX)

S5 Fig Change in nucleosome occupancy at TSSs upon Rsc3 and/or PolyA/T deletion. **A)** The format of the figure is the same as the main Fig 6 except that we either eliminated Rsc3 (*left*), or Rsc3 and PolyA/T (*middle*), or Rsc3 and remodeling effect (*right*) in the model. **B)** Pearson correlation coefficient, R, between experimental [49] and simulated nucleosome occupancy change. Note that the correlation is higher when only Rsc3 is deleted.
(PPTX)

S6 Fig Heatmap of the occupancy of the top 30 TFs and the five PolyA factors on the *D*. *hansenii* YAC introduced into *S*. *cerevisiae*. The top panel shows the occupancy near the TSSs of the 154 genes in the YAC [50]; the lower panel shows the occupancy in fortuitous NDRs generated in the gene body.
(PPTX)

S7 Fig Comparison with other models using Oberbeckmann et al. dataset as the reference. Model performance measured by *ρ*_N_, RMSD, *P*_NDR_, and AUC are compared among different models: N2, Nupop, Dnabend, Segal, Ozonov, and our own model.
(PPTX)

S8 Fig NDR prediction for various annotated NDRs. **A)** NDR Prediction of our model in comparison to various annotated NDRs. The NDRs of Chereji [44] and Yadon [59] are same as in S2E Fig, except this time if the size between the centers of consecutive NDRs is less than 125 bp, they are merged as a single NDR. Oberbeckmann’s [31] NDRs are annotated using the same scheme as Lee’s [6] NDRs (see Materials and Methods). The lists of NDRs and their coordinates for Lee and Oberbeckmann are given in S5 and S6 Tables, respectively. **B)** NDR Prediction of our model for common NDRs in multiple datasets.
(PPTX)

S1 Table Occupancy of the top 30 TFs and the five PolyA factors near TSSs. (XLSX)

S2 Table Occupancy of the top 30 TFs near TTSs the five PolyA factors. (XLSX)

S3 Table Occupancy of the top 30 TFs and the five PolyA factors within gene bodies. (XLSX)

S4 Table List of model parameters. (XLSX)

S5 Table Annotated NDRs (Lee et al.). (XLSX)

S6 Table Annotated NDRs (Oberbeckmann et al.). (XLSX)
