# Supplementary material for: Thermodynamic modeling of genome-wide nucleosome depleted regions in yeast
Source: PLoS Comput Biol. 2021 Jan 11;17(1):e1008560. doi: 10.1371/journal.pcbi.1008560 (PMC7822557; doi:10.1371/journal.pcbi.1008560)
Supplement: S1 Fig — A) Histograms of nucleosome occupancy: Lee et al. [6], Kaplan et al. [3], Mavrich et al. [29], and Oberbeckmann et al. [31]. B) Heatmap of the root-mean-square-deviation (RMSD) between different datasets. C) Composite plot of the average nucleosome occupancy near transcription start sites (TSSs, left) or transcription termination sites (TTSs, right). (PPTX) [file pcbi.1008560.s001.pptx]

## Slide 1
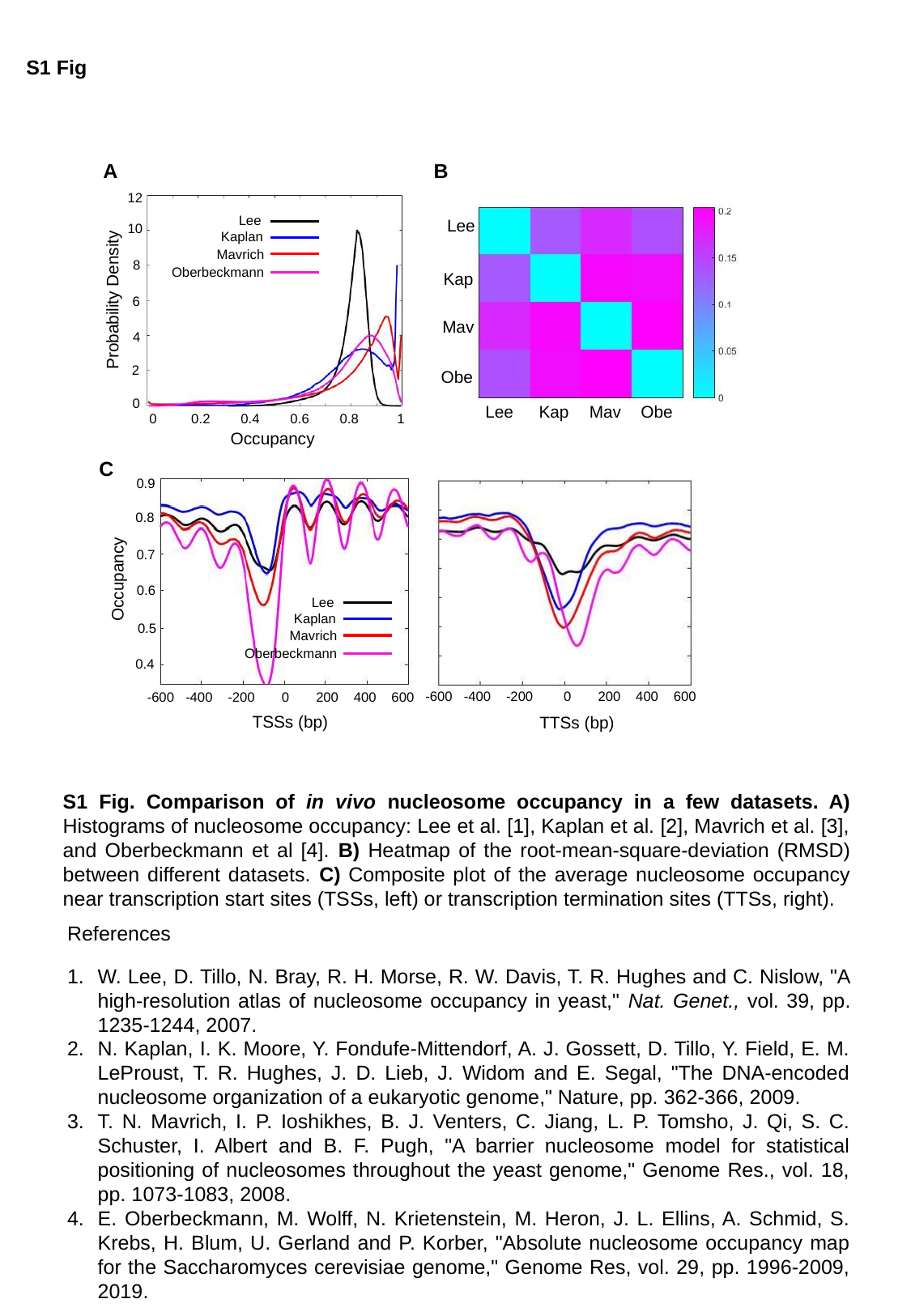

S1 Fig
A
B
12
Lee
Kaplan
Mavrich
Oberbeckmann
Lee
10
8
Kap
Probability Density
6
Mav
4
2
Obe
0
Kap
Lee
Obe
Mav
0 0.2 0.4 0.6 0.8 1
Occupancy
C
0.9
0.8
0.7
Occupancy
0.6
Lee
Kaplan
Mavrich
Oberbeckmann
0.5
0.4
-600 -400 -200 0 200 400 600
-600 -400 -200 0 200 400 600
TSSs (bp)
TTSs (bp)
S1 Fig. Comparison of in vivo nucleosome occupancy in a few datasets. A) Histograms of nucleosome occupancy: Lee et al. [1], Kaplan et al. [2], Mavrich et al. [3], and Oberbeckmann et al [4]. B) Heatmap of the root-mean-square-deviation (RMSD) between different datasets. C) Composite plot of the average nucleosome occupancy near transcription start sites (TSSs, left) or transcription termination sites (TTSs, right).
References
W. Lee, D. Tillo, N. Bray, R. H. Morse, R. W. Davis, T. R. Hughes and C. Nislow, "A high-resolution atlas of nucleosome occupancy in yeast," Nat. Genet., vol. 39, pp. 1235-1244, 2007.
N. Kaplan, I. K. Moore, Y. Fondufe-Mittendorf, A. J. Gossett, D. Tillo, Y. Field, E. M. LeProust, T. R. Hughes, J. D. Lieb, J. Widom and E. Segal, "The DNA-encoded nucleosome organization of a eukaryotic genome," Nature, pp. 362-366, 2009.
T. N. Mavrich, I. P. Ioshikhes, B. J. Venters, C. Jiang, L. P. Tomsho, J. Qi, S. C. Schuster, I. Albert and B. F. Pugh, "A barrier nucleosome model for statistical positioning of nucleosomes throughout the yeast genome," Genome Res., vol. 18, pp. 1073-1083, 2008.
E. Oberbeckmann, M. Wolff, N. Krietenstein, M. Heron, J. L. Ellins, A. Schmid, S. Krebs, H. Blum, U. Gerland and P. Korber, "Absolute nucleosome occupancy map for the Saccharomyces cerevisiae genome," Genome Res, vol. 29, pp. 1996-2009, 2019.
