# Supplementary material for: Thermodynamic modeling of genome-wide nucleosome depleted regions in yeast
Source: PLoS Comput Biol. 2021 Jan 11;17(1):e1008560. doi: 10.1371/journal.pcbi.1008560 (PMC7822557; doi:10.1371/journal.pcbi.1008560)
Supplement: S2 Fig — A) A snapshot of Lee et al. occupancy data [6] showing three examples of potential NDRs: (a), (b), and (c). The occupancy data is discretized using horizontal lines at 80%, 73.2%, 66.4%, etc. with a constant step size of 6.78%. B) Zoom-in views of the annotated NDRs, (a)–(c), in A. “dx” is the distance between a cut-point at 80% line and the lowest crossing points. “l” is the distance between cross points with the 73.2% line. The occupancy was modified so that the occupancies between the lowest cross points are set to 0. The size of NDR is indicated by the red lines (length crossed at the 66.4% line). C) A histogram of dx. For NDR annotation, we require dx to be less than 100bp. D) Modified occupancy in an example region with the sharp downward spikes to 0 occupancy representing NDRs. The original data is shown in blue. The different asterisk points represent locations of annotated NDRs from previous studies: Chereji et al. [44], Yadon et al. [59], and Jiang & Pugh [64]. E) A table showing the number of overlapping NDRs between our annotation and those from Chereji et al. and Yadon et al. (PPTX) [file pcbi.1008560.s002.pptx]

## Slide 1
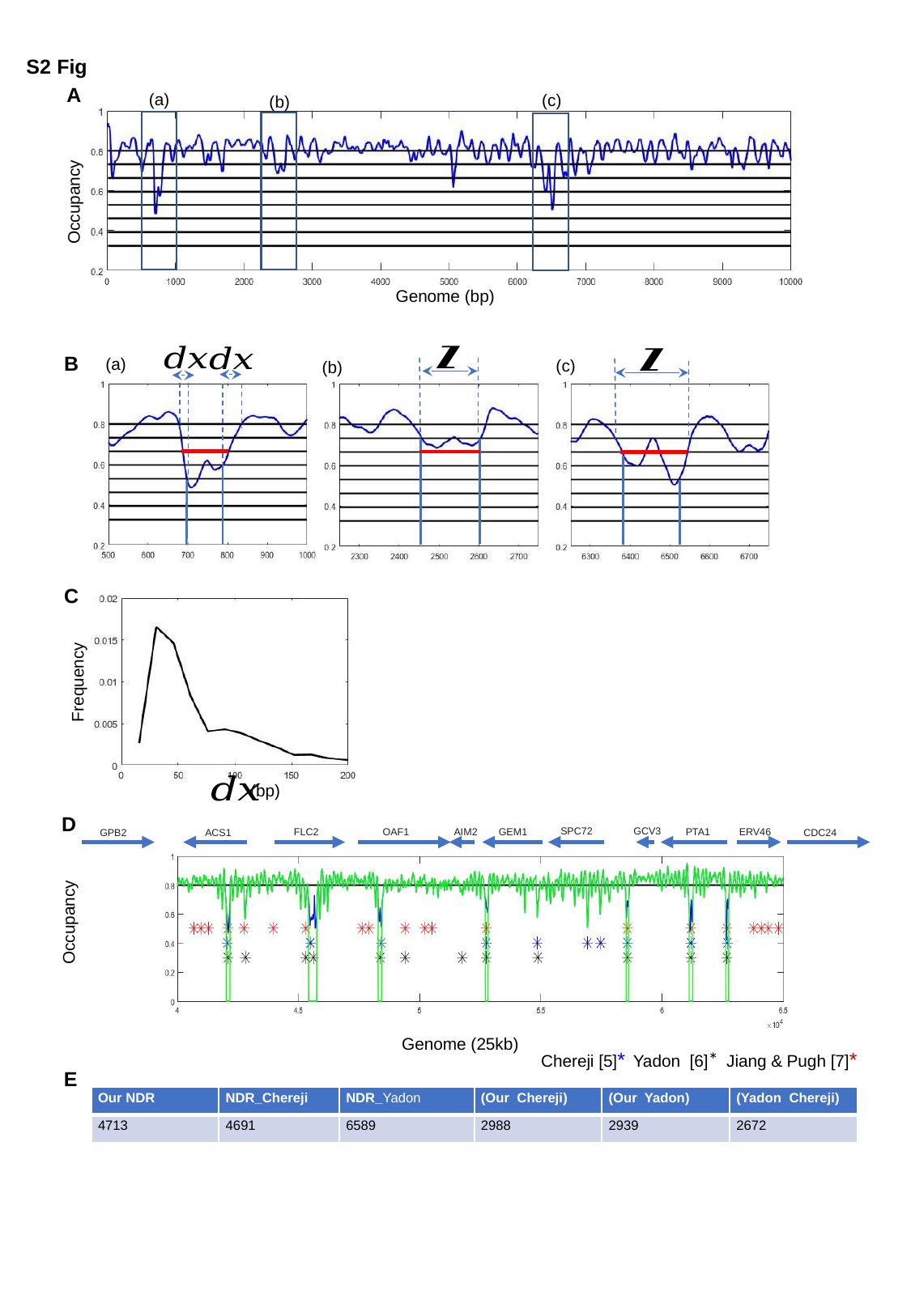

S2 Fig
A
(a)
(c)
(b)
Occupancy
Genome (bp)
B
(a)
(c)
(b)
C
Frequency
 (bp)
D
GCV3
SPC72
ERV46
GEM1
PTA1
FLC2
OAF1
AIM2
CDC24
ACS1
GPB2
Occupancy
Genome (25kb)
Jiang & Pugh [7]*
Yadon [6]*
Chereji [5]*
E

## Slide 2
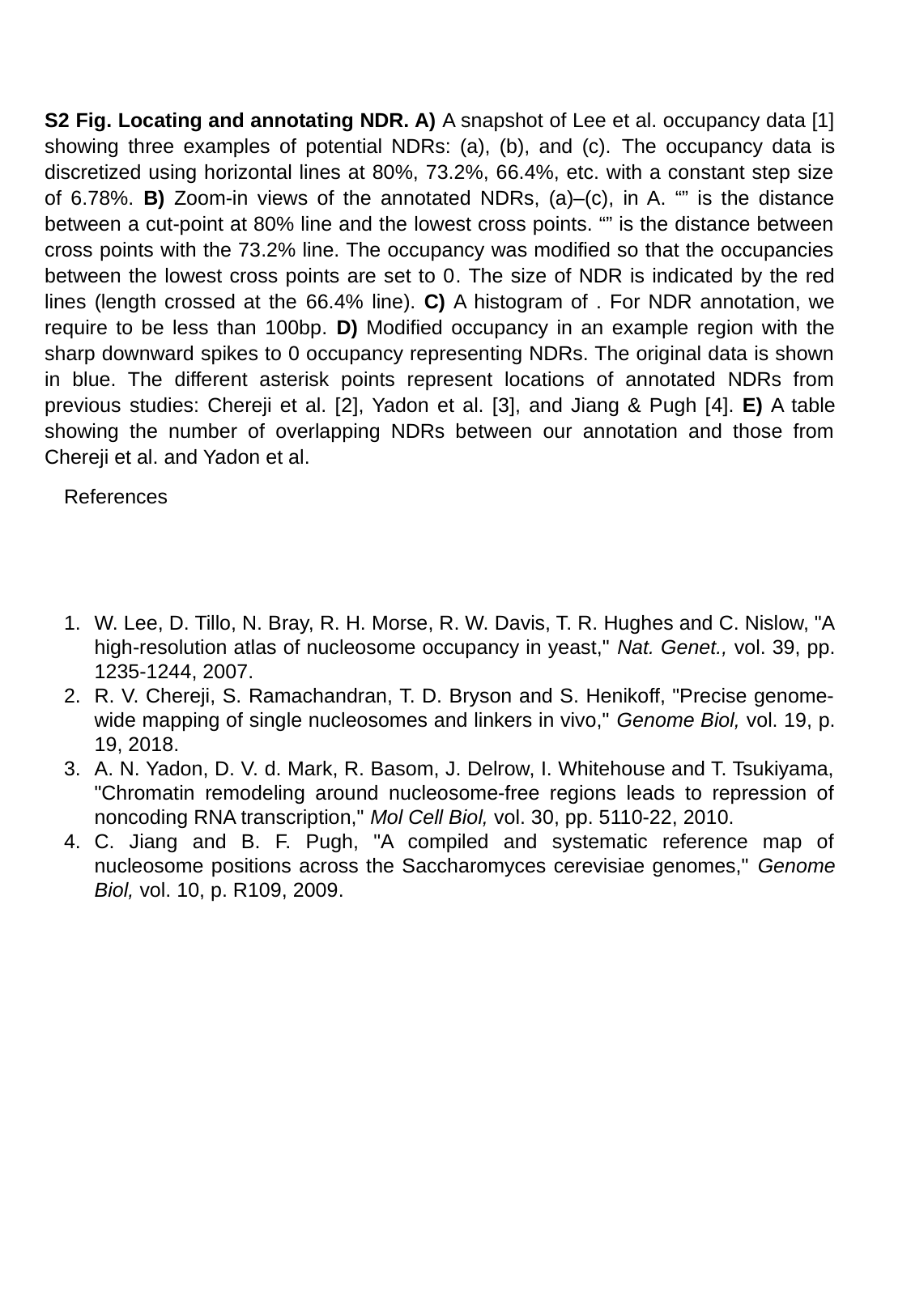

References
W. Lee, D. Tillo, N. Bray, R. H. Morse, R. W. Davis, T. R. Hughes and C. Nislow, "A high-resolution atlas of nucleosome occupancy in yeast," Nat. Genet., vol. 39, pp. 1235-1244, 2007.
R. V. Chereji, S. Ramachandran, T. D. Bryson and S. Henikoff, "Precise genome-wide mapping of single nucleosomes and linkers in vivo," Genome Biol, vol. 19, p. 19, 2018.
A. N. Yadon, D. V. d. Mark, R. Basom, J. Delrow, I. Whitehouse and T. Tsukiyama, "Chromatin remodeling around nucleosome-free regions leads to repression of noncoding RNA transcription," Mol Cell Biol, vol. 30, pp. 5110-22, 2010.
C. Jiang and B. F. Pugh, "A compiled and systematic reference map of nucleosome positions across the Saccharomyces cerevisiae genomes," Genome Biol, vol. 10, p. R109, 2009.
