# Supplementary material for: Thermodynamic modeling of genome-wide nucleosome depleted regions in yeast
Source: PLoS Comput Biol. 2021 Jan 11;17(1):e1008560. doi: 10.1371/journal.pcbi.1008560 (PMC7822557; doi:10.1371/journal.pcbi.1008560)
Supplement: S3 Fig — A) TF cluster density in hit-, missed-, and non-NDRs with different number of TFs taken into consideration (maximum distance between TFs in a cluster, dx, is 12bp). B) Histograms for the number of TFs in a cluster (left) and the size of TF clusters (right) with dx = 147bp. C) TF cluster density in hit-, missed-, and non-NDRs with dx = 147bp. D) Occurrence frequency for Top30 TF motifs in hit-, missed-, and non-NDRs. (PPTX) [file pcbi.1008560.s003.pptx]

## Slide 1
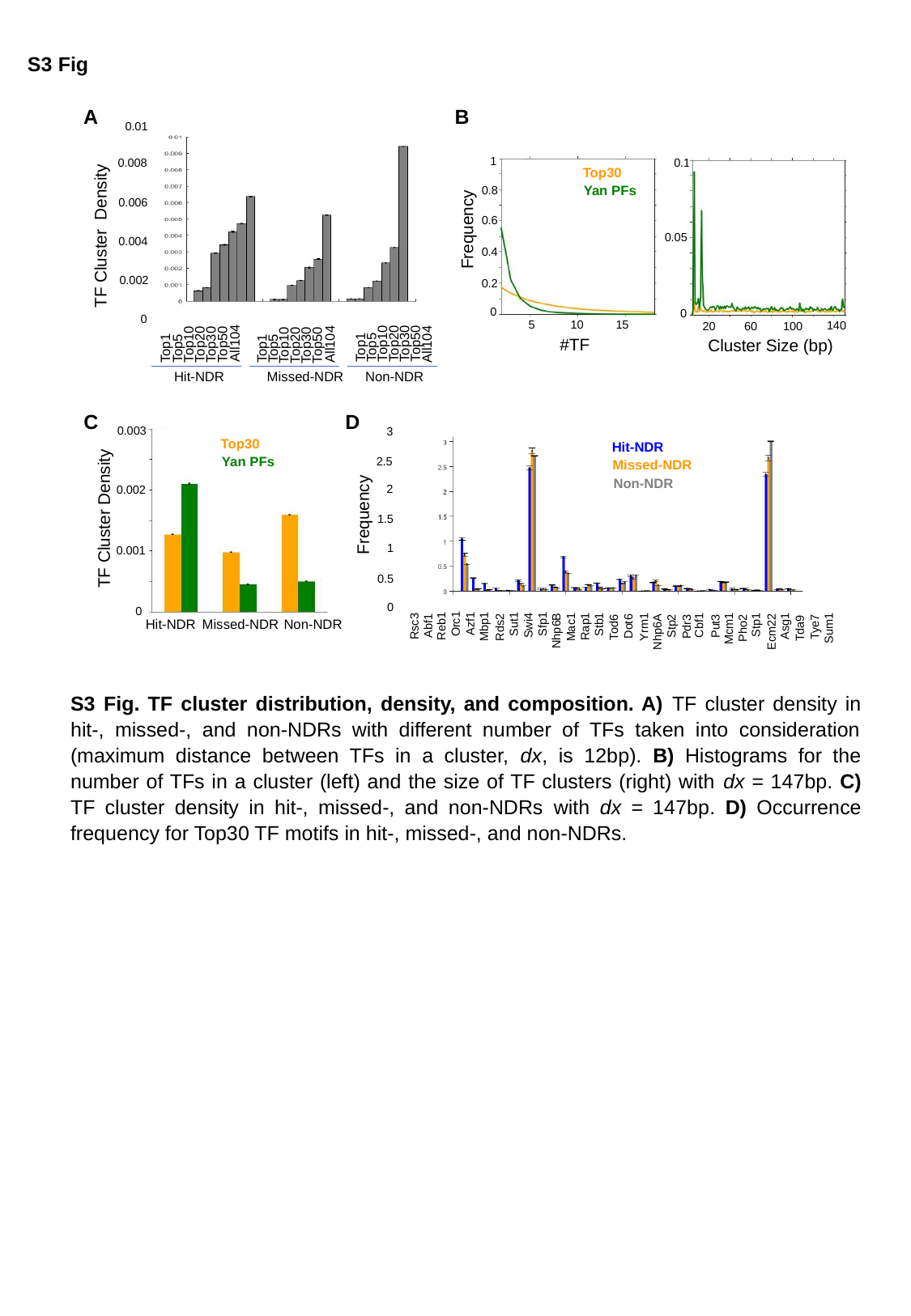

S3 Fig
B
A
0.01
1
0.1
0.008
Top30
Yan PFs
0.8
0.006
0.6
TF Cluster Density
Frequency
0.05
0.004
0.4
0.002
0.2
0
0
0
 5 10 15
140
100
60
20
#TF
Cluster Size (bp)
All104
Top10
Top20
Top50
Top30
Top10
All104
All104
Top20
Top50
Top30
Top10
Top20
Top50
Top30
Top1
Top5
Top1
Top5
Top1
Top5
Hit-NDR
Missed-NDR
Non-NDR
C
D
0.003
3
Top30
Hit-NDR
Yan PFs
2.5
Missed-NDR
Non-NDR
2
0.002
TF Cluster Density
Frequency
1.5
1
0.001
0.5
0
0
Stp1
Stb1
Cbf1
Abf1
Yrm1
Stp2
Mcm1
Mac1
Rap1
Pdr3
Pho2
Missed-NDR
Hit-NDR
Non-NDR
Azf1
Orc1
Sut1
Sfp1
Swi4
Put3
Asg1
Sum1
Reb1
Dot6
Rsc3
Mbp1
Tye7
Rds2
Tod6
Tda9
Nhp6A
Nhp6B
Ecm22
S3 Fig. TF cluster distribution, density, and composition. A) TF cluster density in hit-, missed-, and non-NDRs with different number of TFs taken into consideration (maximum distance between TFs in a cluster, dx, is 12bp). B) Histograms for the number of TFs in a cluster (left) and the size of TF clusters (right) with dx = 147bp. C) TF cluster density in hit-, missed-, and non-NDRs with dx = 147bp. D) Occurrence frequency for Top30 TF motifs in hit-, missed-, and non-NDRs.
