# Supplementary material for: Thermodynamic modeling of genome-wide nucleosome depleted regions in yeast
Source: PLoS Comput Biol. 2021 Jan 11;17(1):e1008560. doi: 10.1371/journal.pcbi.1008560 (PMC7822557; doi:10.1371/journal.pcbi.1008560)
Supplement: S4 Fig — Heatmap of the occupancy of the top 30 TFs and the five PolyA factors near TSSs (top), within gene bodies (center), and near TTSs (bottom). We have listed all 5542 genes with the exact TSS and TTS coordinates, and corresponding values can be found in S1, S2 and S3 Tables. The gene indices and annotations are adapted from ref. [44]. (PPTX) [file pcbi.1008560.s004.pptx]

## Slide 1
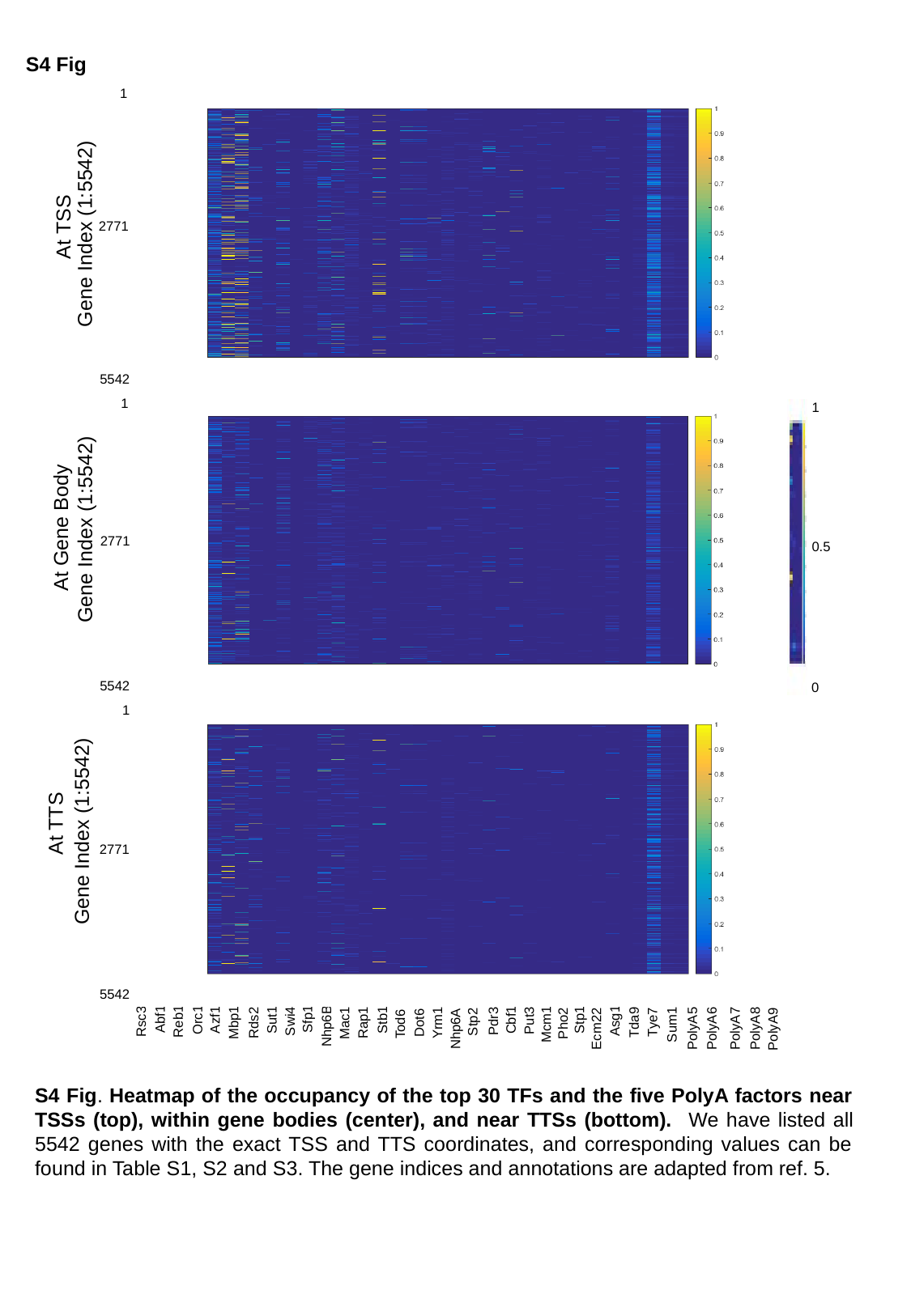

S4 Fig
1
At TSS
2771
Gene Index (1:5542)
5542
1
1
At Gene Body
Gene Index (1:5542)
2771
0.5
5542
0
1
At TTS
Gene Index (1:5542)
2771
5542
Stp2
Azf1
Orc1
Stb1
Abf1
Pdr3
Rsc3
 Sfp1
Swi4
Rap1
Dot6
Mbp1
Mac1
Tod6
Reb1
Cbf1
Sut1
Rds2
Put3
PolyA5
PolyA6
PolyA8
PolyA7
Nhp6A
Sum1
PolyA9
Tye7
Yrm1
Mcm1
Nhp6B
Asg1
Stp1
Ecm22
Tda9
Pho2
S4 Fig. Heatmap of the occupancy of the top 30 TFs and the five PolyA factors near TSSs (top), within gene bodies (center), and near TTSs (bottom). We have listed all 5542 genes with the exact TSS and TTS coordinates, and corresponding values can be found in Table S1, S2 and S3. The gene indices and annotations are adapted from ref. 5.
