# Supplementary material for: Thermodynamic modeling of genome-wide nucleosome depleted regions in yeast
Source: PLoS Comput Biol. 2021 Jan 11;17(1):e1008560. doi: 10.1371/journal.pcbi.1008560 (PMC7822557; doi:10.1371/journal.pcbi.1008560)
Supplement: S5 Fig — A) The format of the figure is the same as the main Fig 6 except that we either eliminated Rsc3 (left), or Rsc3 and PolyA/T (middle), or Rsc3 and remodeling effect (right) in the model. B) Pearson correlation coefficient, R, between experimental [49] and simulated nucleosome occupancy change. Note that the correlation is higher when only Rsc3 is deleted. (PPTX) [file pcbi.1008560.s005.pptx]

## Slide 1
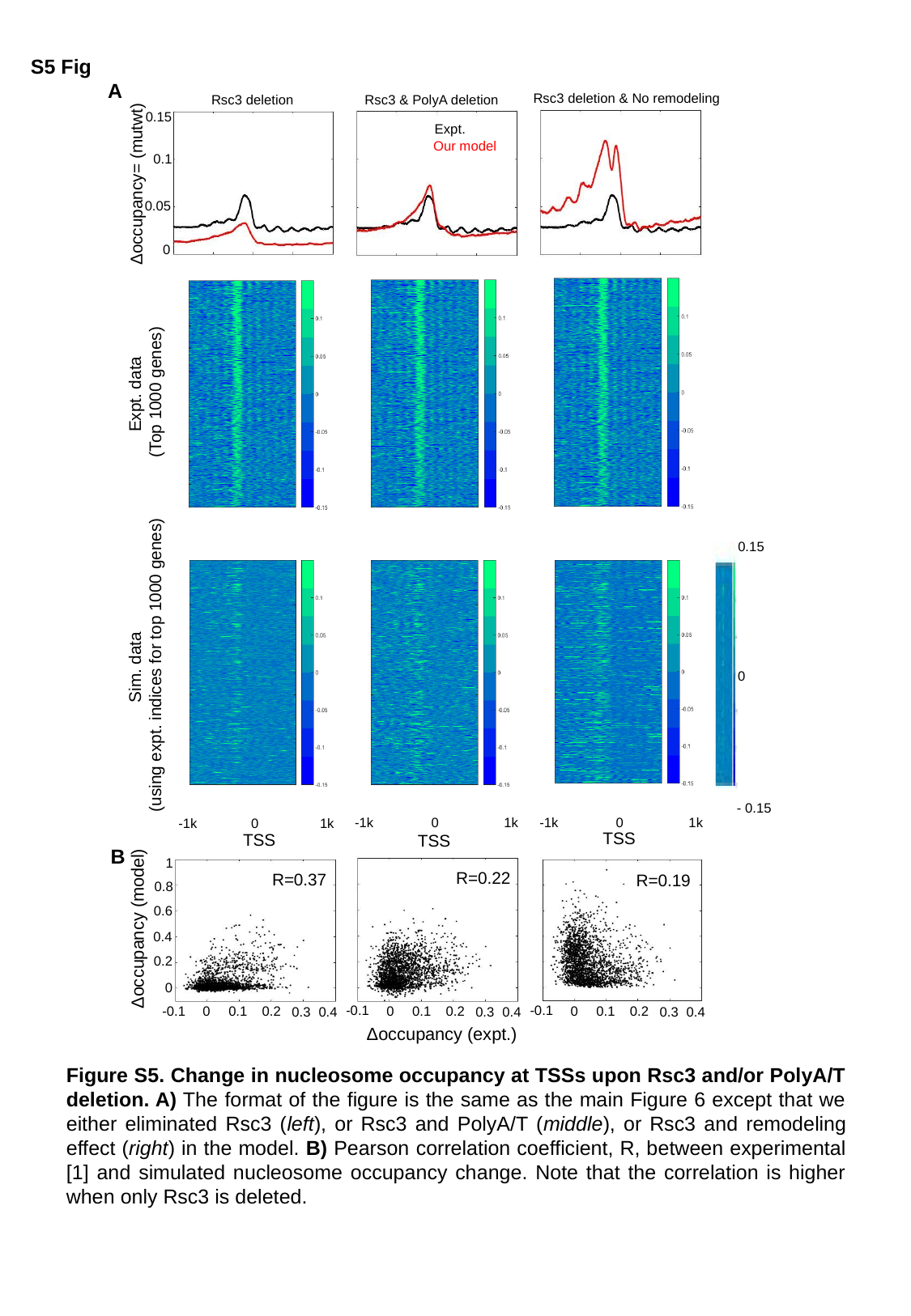

S5 Fig
A
Rsc3 deletion & No remodeling
Rsc3 & PolyA deletion
Rsc3 deletion
0.15
Expt.
Our model
0.1
0.05
0
Expt. data
(Top 1000 genes)
0.15
Sim. data
(using expt. indices for top 1000 genes)
0
- 0.15
 -1k 0 1k
 -1k 0 1k
 -1k 0 1k
TSS
TSS
TSS
B
1
R=0.22
R=0.37
0.8
0.6
Δoccupancy (model)
0.4
0.2
0
-0.1
-0.1
-0.1
0
0.1
0.2
0
0.1
0.2
0
0.1
0.2
0.3
0.4
0.3
0.4
0.3
0.4
Δoccupancy (expt.)
Figure S5. Change in nucleosome occupancy at TSSs upon Rsc3 and/or PolyA/T deletion. A) The format of the figure is the same as the main Figure 6 except that we either eliminated Rsc3 (left), or Rsc3 and PolyA/T (middle), or Rsc3 and remodeling effect (right) in the model. B) Pearson correlation coefficient, R, between experimental [1] and simulated nucleosome occupancy change. Note that the correlation is higher when only Rsc3 is deleted.

## Slide 2
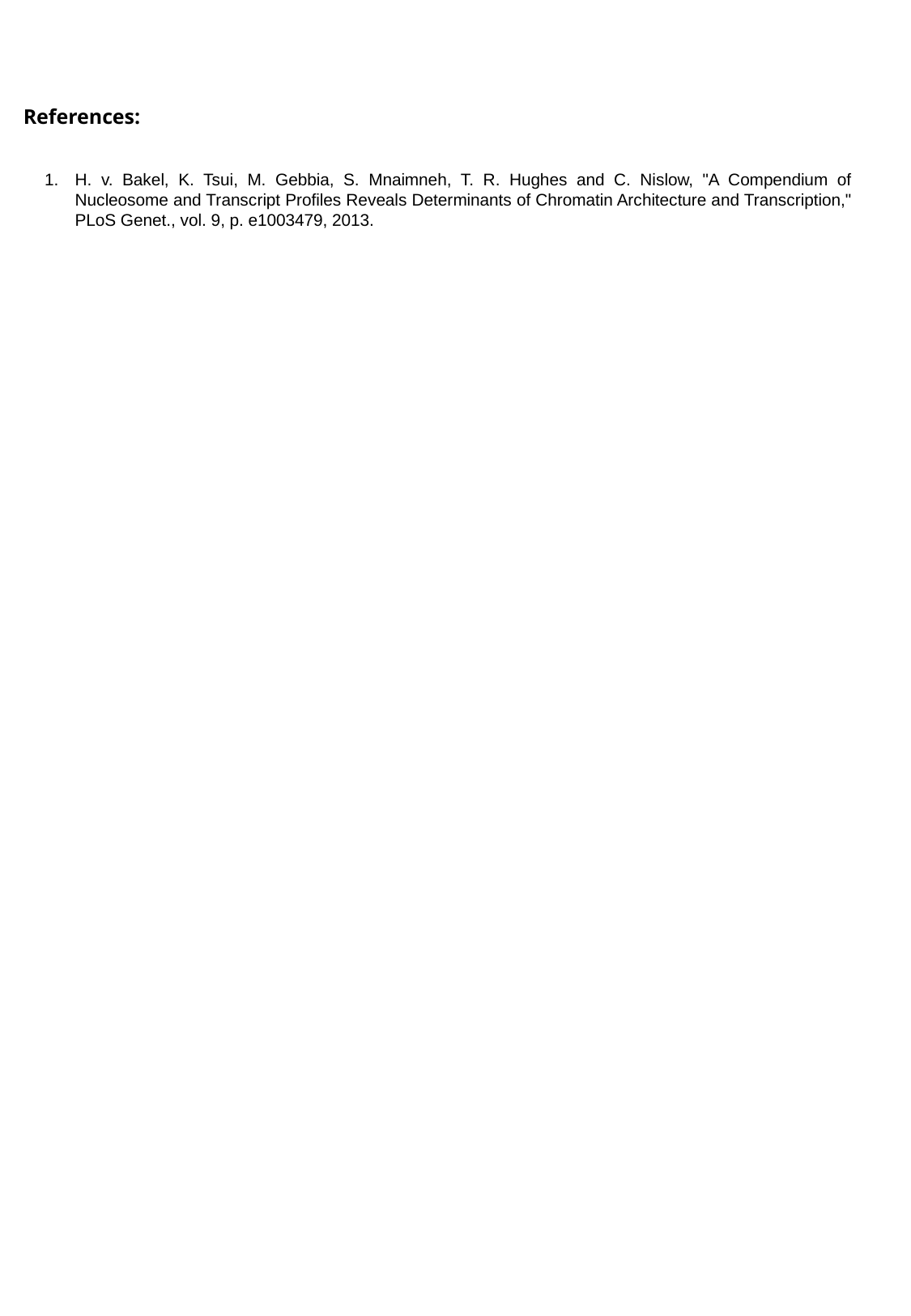

References:
H. v. Bakel, K. Tsui, M. Gebbia, S. Mnaimneh, T. R. Hughes and C. Nislow, "A Compendium of Nucleosome and Transcript Profiles Reveals Determinants of Chromatin Architecture and Transcription," PLoS Genet., vol. 9, p. e1003479, 2013.
