# Supplementary material for: Thermodynamic modeling of genome-wide nucleosome depleted regions in yeast
Source: PLoS Comput Biol. 2021 Jan 11;17(1):e1008560. doi: 10.1371/journal.pcbi.1008560 (PMC7822557; doi:10.1371/journal.pcbi.1008560)
Supplement: S6 Fig — The top panel shows the occupancy near the TSSs of the 154 genes in the YAC [50]; the lower panel shows the occupancy in fortuitous NDRs generated in the gene body. (PPTX) [file pcbi.1008560.s006.pptx]

## Slide 1
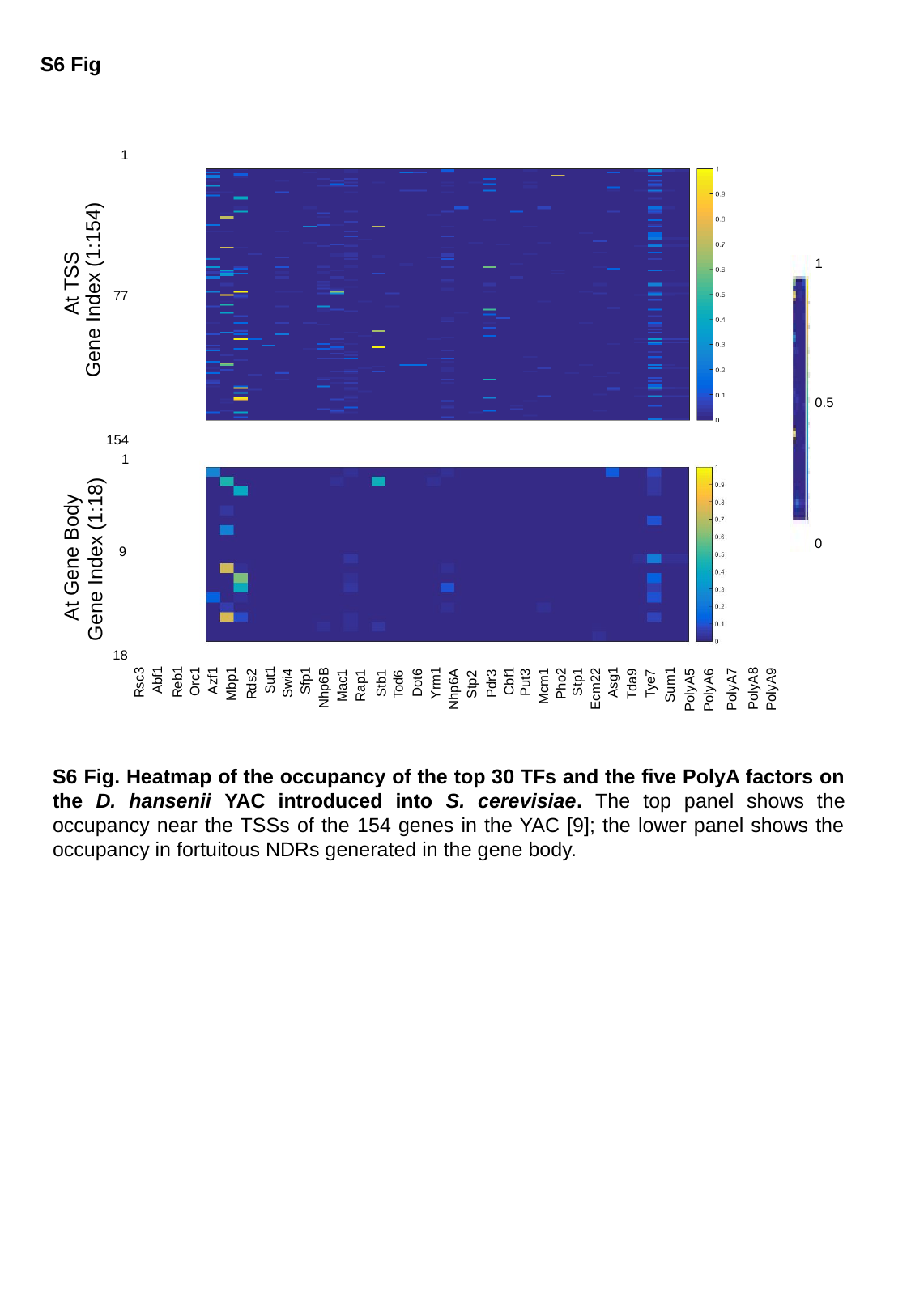

S6 Fig
1
1
At TSS
Gene Index (1:154)
77
0.5
154
1
0
9
At Gene Body
Gene Index (1:18)
18
Stp2
Azf1
Orc1
Abf1
Rsc3
Stb1
Dot6
 Sfp1
Pdr3
Mbp1
Swi4
Tod6
Mac1
Rap1
Reb1
Cbf1
Sut1
Asg1
PolyA8
Nhp6A
Sum1
PolyA7
PolyA9
Rds2
PolyA5
PolyA6
Yrm1
Put3
Tye7
Mcm1
Nhp6B
Ecm22
Stp1
Pho2
Tda9
S6 Fig. Heatmap of the occupancy of the top 30 TFs and the five PolyA factors on the D. hansenii YAC introduced into S. cerevisiae. The top panel shows the occupancy near the TSSs of the 154 genes in the YAC [9]; the lower panel shows the occupancy in fortuitous NDRs generated in the gene body.
