# Supplementary material for: Thermodynamic modeling of genome-wide nucleosome depleted regions in yeast
Source: PLoS Comput Biol. 2021 Jan 11;17(1):e1008560. doi: 10.1371/journal.pcbi.1008560 (PMC7822557; doi:10.1371/journal.pcbi.1008560)
Supplement: S7 Fig — Model performance measured by ρN, RMSD, PNDR, and AUC are compared among different models: N2, Nupop, Dnabend, Segal, Ozonov, and our own model. (PPTX) [file pcbi.1008560.s007.pptx]

## Slide 1
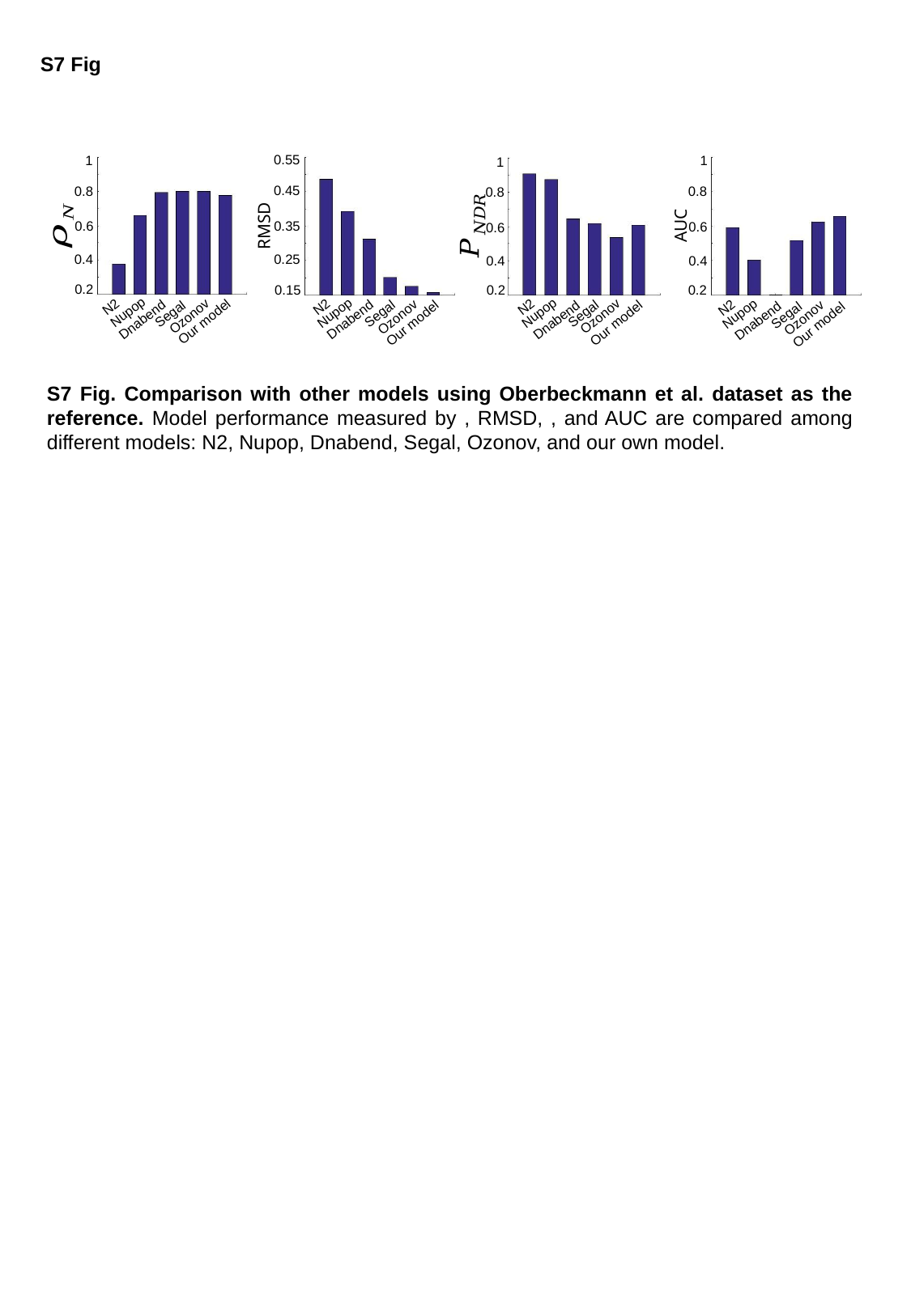

S7 Fig
0.55
1
1
1
0.45
0.8
0.8
0.8
AUC
RMSD
0.6
0.35
0.6
0.6
0.4
0.25
0.4
0.4
0.2
0.15
0.2
0.2
N2
N2
N2
N2
Nupop
Nupop
Nupop
Segal
Segal
Nupop
Segal
Segal
Ozonov
Ozonov
Ozonov
Ozonov
Dnabend
Dnabend
Dnabend
Dnabend
Our model
Our model
Our model
Our model
