# Supplementary material for: Thermodynamic modeling of genome-wide nucleosome depleted regions in yeast
Source: PLoS Comput Biol. 2021 Jan 11;17(1):e1008560. doi: 10.1371/journal.pcbi.1008560 (PMC7822557; doi:10.1371/journal.pcbi.1008560)
Supplement: S8 Fig — A) NDR Prediction of our model in comparison to various annotated NDRs. The NDRs of Chereji [44] and Yadon [59] are same as in S2E Fig, except this time if the size between the centers of consecutive NDRs is less than 125 bp, they are merged as a single NDR. Oberbeckmann’s [31] NDRs are annotated using the same scheme as Lee’s [6] NDRs (see Materials and Methods). The lists of NDRs and their coordinates for Lee and Oberbeckmann are given in S5 and S6 Tables, respectively. B) NDR Prediction of our model for common NDRs in multiple datasets. (PPTX) [file pcbi.1008560.s008.pptx]

## Slide 1
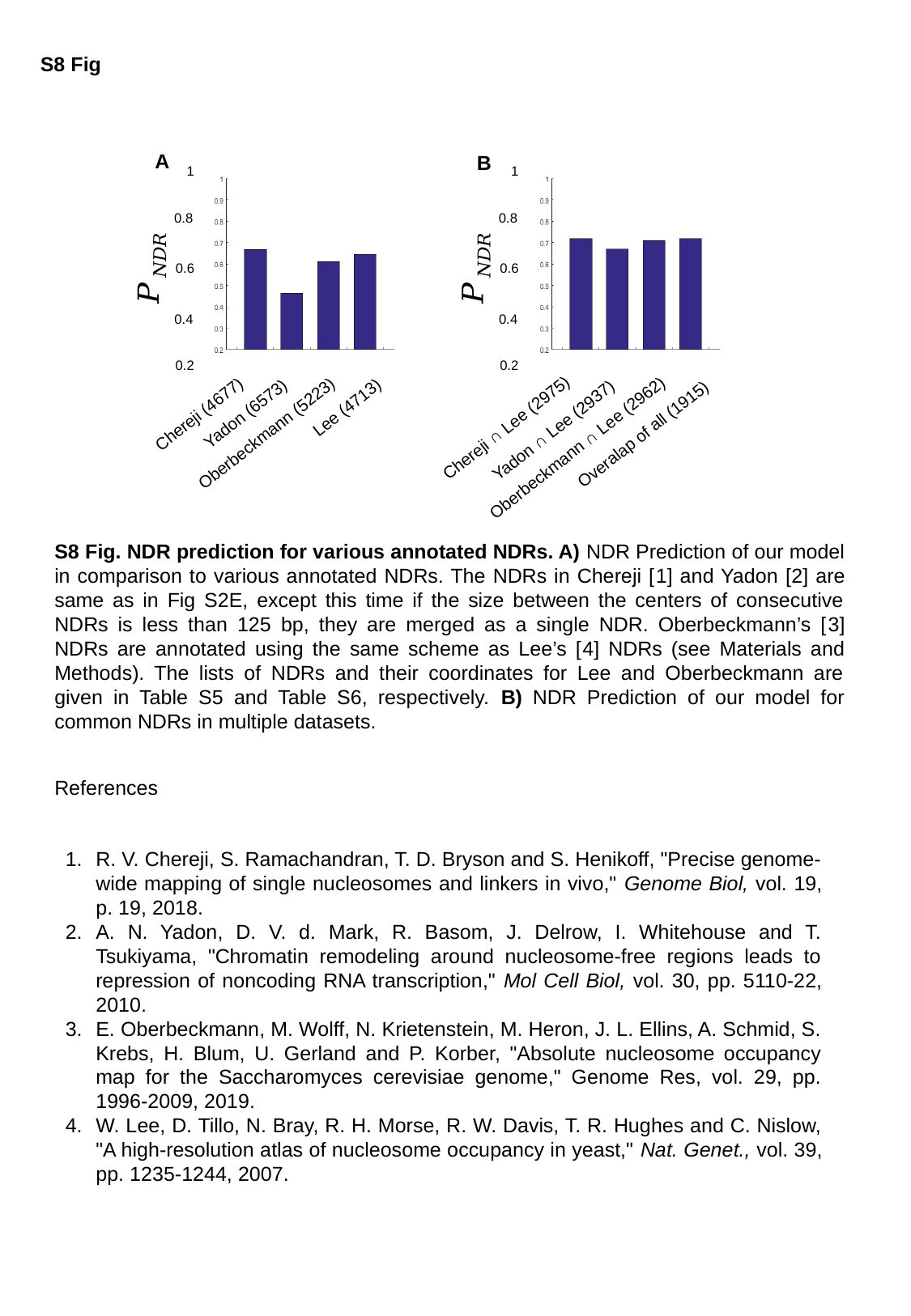

S8 Fig
A
B
1
1
0.8
0.8
0.6
0.6
0.4
0.4
0.2
0.2
Lee (4713)
Yadon (6573)
Chereji (4677)
Chereji ∩ Lee (2975)
Yadon ∩ Lee (2937)
Oberbeckmann (5223)
Overalap of all (1915)
Oberbeckmann ∩ Lee (2962)
S8 Fig. NDR prediction for various annotated NDRs. A) NDR Prediction of our model in comparison to various annotated NDRs. The NDRs in Chereji [1] and Yadon [2] are same as in Fig S2E, except this time if the size between the centers of consecutive NDRs is less than 125 bp, they are merged as a single NDR. Oberbeckmann’s [3] NDRs are annotated using the same scheme as Lee’s [4] NDRs (see Materials and Methods). The lists of NDRs and their coordinates for Lee and Oberbeckmann are given in Table S5 and Table S6, respectively. B) NDR Prediction of our model for common NDRs in multiple datasets.
References
R. V. Chereji, S. Ramachandran, T. D. Bryson and S. Henikoff, "Precise genome-wide mapping of single nucleosomes and linkers in vivo," Genome Biol, vol. 19, p. 19, 2018.
A. N. Yadon, D. V. d. Mark, R. Basom, J. Delrow, I. Whitehouse and T. Tsukiyama, "Chromatin remodeling around nucleosome-free regions leads to repression of noncoding RNA transcription," Mol Cell Biol, vol. 30, pp. 5110-22, 2010.
E. Oberbeckmann, M. Wolff, N. Krietenstein, M. Heron, J. L. Ellins, A. Schmid, S. Krebs, H. Blum, U. Gerland and P. Korber, "Absolute nucleosome occupancy map for the Saccharomyces cerevisiae genome," Genome Res, vol. 29, pp. 1996-2009, 2019.
W. Lee, D. Tillo, N. Bray, R. H. Morse, R. W. Davis, T. R. Hughes and C. Nislow, "A high-resolution atlas of nucleosome occupancy in yeast," Nat. Genet., vol. 39, pp. 1235-1244, 2007.
